# The nonlinear porous medium equation for the *f*-Laplacian: Hamilton-Souplet-Zhang type gradient estimates and implications

**DOI:** 10.1007/s10231-025-01639-z

**Published:** 2025-11-24

**Authors:** Ali Taheri, Vahideh Vahidifar

**Affiliations:** School of Mathematical and Physical Sciences, University of Sussex, Falmer, Brighton UK

**Keywords:** Smooth metric measure spaces, *f*-Laplacian, Nonlinear diffusion, Porous medium equation, Super Perelman-Ricci flow, Hamilton-Souplet-Zhang estimates, Ancient solutions, 53C44, 58J60, 58J35, 60J60

## Abstract

This article presents new gradient estimates for positive solutions to the nonlinear porous medium equation in the context of smooth metric measure spaces. The diffusion operator here is the *f*-Laplacian and the gradient estimates of interest are mainly of Hamilton-Souplet-Zhang types. These estimates are established using a variety of methods and techniques and several implications, most notably, to parabolic Liouville-type results and characterisation of ancient solutions are given. The problem is posed in the general framework where the metric and potential evolve with time and the proofs make use of natural lower bounds on the time derivative of the metric and the Bakry-Émery *m*-Ricci curvature tensors. Our results extend and improve various existing ones in the literature.

## Introduction

In this paper we are primarily concerned with nonlinear diffusion equations of porous medium type, or equivalently, slow diffusion type, in the framework of smooth metric measure spaces (hereafter we write SMMS for brevity). The motivation for this study is prompted by the recent surge of interest in understanding the analytic and dynamic behaviour of solutions, the probabilistic features of diffusion on SMMS and the role that geometry and curvature on the one hand and nonlinearity on the other play in forming such behaviour and features (*see*, e.g. [1, 7, 9–11, 14, 30, 31, 44, 45, 47]).

Towards this end, let $(\mathscr {M}, g)$ be a complete Riemannian manifold of dimension $n \ge 2$, $d\mu =e^{-f} dv_g$ be a positive weighted measure associated with the metric *g* and potential *f* and $dv_g$ be the standard Riemannain volume measure on $\mathscr M$. The triple $(\mathscr M, g, d\mu )$ is referred to as a smooth metric measure space (or a weighted manifold). Our prime interest in this paper is the nonlinear porous medium equation associated with the triple $(\mathscr M,g,d\mu )$ that can be written for a positive $u=u(x,t)$ in the form:

1.1 $$\begin{aligned} \square _p u(x,t) = \partial _t u (x,t) - \Delta _f u^p (x,t) = {\mathscr {N}}(t,x,u(x,t)), \qquad p>1. \end{aligned}$$

The operator $\Delta _f$ in (1.1) is the *f*-Laplacian (also called Witten or weighted Laplacian) that acts on functions $w \in \mathscr {C}^2(\mathscr M)$ as

1.2 $$\begin{aligned} \Delta _f w = e^f \textrm{div}(e^{-f} \nabla w) = \Delta w - \langle \nabla f, \nabla w\rangle . \end{aligned}$$

The exponent $p>1$ is a fixed constant and ${{\mathscr {N}}}={{\mathscr {N}}}(t,x,u)$ is a sufficiently smooth *nonlinearity* or forcing term that depends on the space-time variables (*x*, *t*) and the dependent variable $u>0$. The structural features of this nonlinearity have analytical and physical significances and one of our aims is to understand the ways it influences the estimates and subsequently the solutions. Our task is to develop gradient estimates of elliptic and Hamilton-Souplet-Zhang types for the positive solutions $u=u(x,t)$ to (1.1) where we use a variety of techniques and ideas to deal with different exponent ranges and investigate some of their important implications.

The porous medium equation (PME) arises in many contexts with many applications. Indeed for various values of $p>1$ it arises in different models of diffusive phenomenon: Boussinesq’s model of ground water infiltration, flow of gas in porous media, heat radiation in plasma, liquid thin films and so on. We refer the interested reader to the monographs and texts [4, 14, 27, 43] and the references therein for a comprehensive coverage of these applications and the underlying theory. The PME can be seen as the simplest generalisation of the linear heat equation to the nonlinear context (coinciding with the latter when $p=1$). The use of terminology *slow diffusion* here is intertwined with the exponent range $p>1$ in (1.1), where, due to the density dependent diffusion coefficient vanishing at $u=0$ – as can be seen by writing $\Delta _f u^p=pe^f \textrm{div} (e^{-f} u^{p-1} \nabla u)$ – disturbances of $u=0$ propagate forward in time with finite speed. This is in sharp contrast to the linear heat as well as the fast (and very fast) diffusion case $0<p<1$ that has a completely different nature and morphology and will be treated in a separate paper.

Following standard theory (*see* [4, 14, 43]), we consider in the SMMS context under study here, the second order linear (space-time dependent variable coefficient) evolution operator defined by

1.3 $$\begin{aligned} {\mathscr {L}}_v^p = \partial _t -(p-1) v(x,t) \Delta _f, \end{aligned}$$

where *v* relates to *u* via the transformation $v =pu^{p-1}/(p-1)$ (the pressure transform) and $p>1$. This operator plays an important role throughout the paper, particularly, in that, if *u* is a positive solution to (1.1), then the pressure *v* is a positive solution to a related equation involving the linear (heat-type) operator ${\mathscr {L}}_v^p$. See Sects. 3 and 7 for more and the discussion surrounding (1.15) at the end of this section.

The *f*-Laplacian $\Delta _f$ is a symmetric diffusion operator with respect to the invariant weighted measure $d\mu$ and arises in a variety of contexts ranging from probability theory and geometry to quantum field theory and statistical mechanics [7, 45]. It is a natural generalisation of the Laplace-Beltrami operator to the SMMS context and it coincides with the latter precisely when the potential *f* is spatially constant. By an application of the integration by parts formula it can be seen that for $u, v \in {{\mathscr {C}}}_0^\infty (\mathscr M)$ it holds

1.4 $$\begin{aligned} \int _{\mathscr M} u \Delta _f v \, d\mu = - \int _{\mathscr M} \langle \nabla u, \nabla v \rangle \, d\mu = \int _{\mathscr M} v \Delta _f u \, d\mu . \end{aligned}$$

As for the geometry and curvature properties of the triple $(\mathscr M,g,d\mu )$ we have a hierarchy of second order symmetric tensor fields on $\mathscr M$ defined by,

1.5 $$\begin{aligned} {\mathscr {R}ic}^m_f(g) := {\mathscr {R}ic}(g) + \nabla \nabla f - \frac{\nabla f \otimes \nabla f}{m-n}, \qquad m \ge n, \end{aligned}$$

called the Bakry-Èmery *m*-Ricci curvature tensors. Here ${\mathscr {R}ic}(g)$ is the usual Riemannain Ricci curvature tensor of *g*, $\nabla \nabla f=\textrm{Hess}(f)$ denotes the Hessian of *f*, and $m \ge n$ is a constant (*see* [7]). For the sake of clarification, in relation to (1.5), when $m=n$, by convention *f* is only allowed to be a constant, thus giving ${\mathscr {R}ic}^m_f(g)={\mathscr {R}ic}(g)$. We also allow for $m = \infty$ in which case by formally passing to the limit in (1.5) we define,

1.6 $$\begin{aligned} {\mathscr {R}ic}_f(g) = {\mathscr {R}ic}(g) + \nabla \nabla f := {\mathscr {R}ic}^\infty _f(g). \end{aligned}$$

By an easy inspection it is seen that having a lower bound on ${\mathscr {R}ic}_f^m(g)$ is a stronger condition than having the same lower bound on ${\mathscr {R}ic}_f(g)$, that is,

1.7 $$\begin{aligned} {\mathscr {R}ic}_f^m(g) \ge {{\textsf{k}}} g \implies {\mathscr {R}ic}_f(g) \ge {{\textsf{k}}} g, \end{aligned}$$

but the reverse implication is not true (here ${{\textsf{k}}} \in {{\mathbb {R}}}$, $m \ge n$ are finite constants). This remark is useful when comparing the results for nonlinear diffusion equations here with the ones for the linear heat-type equations.

The crucial identity relating the *f*-Laplacian $\Delta _f$ to the Bakry-Emery Ricci curvature tensor ${\mathscr {R}ic}_f(g)$ in this context is the weighted Bochner-Weitzenböck formula (generalising, in turn, the classical version of the identity to the context of SMMS, *see*, e.g., [5, 24, 26]) asserting that or any function *w* of class ${{\mathscr {C}}}^3(\mathscr M)$ we have,

1.8 $$\begin{aligned} \frac{1}{2} \Delta _f |\nabla w|^2 - \langle \nabla w, \nabla \Delta _f w \rangle = |\nabla \nabla w|^2 + {\mathscr {R}ic}_f (\nabla w, \nabla w). \end{aligned}$$

While this identity is not immediately applicable to the Bakry-Émery *m*-Ricci curvature tensor, upon adding and subtracting the symmetric second order rank-one tensor $[\nabla f \otimes \nabla f]/(m-n)$ to (1.8), making use of (1.5) and then an application of the Cauchy-Schwarz inequality, one can arrive at a counterpart *inequality* in the form

1.9 $$\begin{aligned} \frac{1}{2} \Delta _f |\nabla w|^2 - \langle \nabla w, \nabla \Delta _f w \rangle&\ge \frac{(\Delta w)^2}{n} + \frac{[\nabla f \otimes \nabla f]}{m-n} (\nabla w, \nabla w) + {\mathscr {R}ic}^m_f (\nabla w, \nabla w) \nonumber \\&\ge \frac{ (\Delta _f w)^2}{m} + {\mathscr {R}ic}^m_f (\nabla w, \nabla w). \end{aligned}$$

Interestingly from (1.8) or (1.9) it follows that subject to a curvature lower bound in the form ${\mathscr {R}ic}_f(g) \ge {{\textsf{k}}} g$ or ${\mathscr {R}ic}^m_f(g) \ge {{\textsf{k}}} g$ with ${{\textsf{k}}} \in {{\mathbb {R}}}$, the diffusion operator $\Delta _f$ satisfies the curvature-dimension condition $\textrm{CD}({{\textsf{k}}}, \infty )$ or $\textrm{CD}({{\textsf{k}}}, m)$ respectively which in turn plays an important role in the study of the dynamics, convergence to equilibrium and the derivation various functional and geometric inequalities associated with $\Delta _f$ (*see*, e.g., [7] for more).

Gradient estimates lie at the core of geometric analysis and have huge applications. For heat and Schrödinger type equations on static manifolds they were first established in the seminal paper of Li and Yau [25]. In the nonlinear setting, equations of heat-type with a logarithmic nonlinearity were among the first to be considered (*see* [23, 28]). In the context of SMMS the nonlinear diffusion version of these equations can be written in the form (1.1) with ${{\mathscr {N}}}(t,x,u)={{\textsf{A}}}(t,x) u \log u$, specifically,

1.10 $$\begin{aligned} \square _p u(x,t) = \frac{\partial u}{\partial t} (x,t) - \Delta _f u^p (x,t) = {{\textsf{A}}}(t,x) u(x,t) \log u(x,t). \end{aligned}$$

The interest in such class of problems come from the natural links with geometric and functional inequalities on manifolds (*cf.* [7, 16, 44, 45, 47] and the references therein for more). Other classes of equations closely relating to (1.10) include

1.11 $$\begin{aligned} \square _p u(x,t) = \frac{\partial u}{\partial t} (x,t) - \Delta _f u^p (x,t) = {{\textsf{A}}}(t,x) u^\alpha (x,t) \Gamma (\log u(x,t)) + {{\textsf{B}}}(t,x) u^\beta (x,t), \end{aligned}$$

with ${{\mathscr {N}}}(t,x,u) = {{\textsf{A}}}(t,x) u^\alpha \Gamma (\log u) + {{\textsf{B}}}(t,x) u^\beta$ where $\alpha$, $\beta$ are real exponents, ${{\textsf{A}}}$, ${{\textsf{B}}}$ are sufficiently smooth space-time functions and $\Gamma \in {{\mathscr {C}}}^1(\mathbb {R}, \mathbb {R})$. Another class of equations that have been extensively studied are Yamabe type equations. In the SMMS context, the nonlinear diffusion version of these equations take the form

1.12 $$\begin{aligned} \square _p u(x,t) = \frac{\partial u}{\partial t} (x,t) - \Delta _f u^p (x,t) = {{\textsf{A}}}(t,x) u^\alpha (x,t) + {{\textsf{B}}}(t,x) u^\beta (x,t). \end{aligned}$$

A far reaching generalisation of (1.12) with a superposition of power-like nonlinearities consist of equations in the form

1.13 $$\begin{aligned} \square _p u(x,t)&= \frac{\partial u}{\partial t} (x,t) - \Delta _f u^p(x,t) \nonumber \\&= \sum _{j=1}^d {{\textsf{A}}}_j(t,x) u^{\alpha _j} (x,t) + \sum _{j=1}^d {{\textsf{B}}}_j(t,x) u^{\beta _j} (x,t). \end{aligned}$$

Here by comparison with (1.1) it is seen that the nonlinearity takes the form

1.14 $$\begin{aligned} {{\mathscr {N}}}(t,x,u) = \sum _{j=1}^d {{\textsf{A}}}_j(t,x) u^{\alpha _j} + \sum _{j=1}^d {{\textsf{B}}}_j(t,x) u^{\beta _j}, \end{aligned}$$

where ${{\textsf{A}}}_j$, ${{\textsf{B}}}_j$ (with $1 \le j \le d$) are sufficiently smooth space-time dependent coefficients and $\alpha _j \ge 0$, $\beta _j \le 0$ are real exponents (*see* [36–38]).

The context in which we consider equation (1.1) is one where the metric tensor *g* and the potential *f* are allowed to be time dependent. Such problems have particularly moved to the forefront of research in geometric analysis since the work of G. Perelman [31] and the study of forward and backward heat-type equations and more generally gradient flows on manifolds evolving under (super) Ricci or (super) Perelman-Ricci flows (*see* also [6, 13, 32, 36–38, 40, 47]).

In this time dependent setting, the measure $d\mu = e^{-f} dv_g$, the Bakry-Émery *m*-Ricci curvature tensor ${\mathscr {R}ic}_f^m(g)$ and the usual (metric dependent) differential operators $\nabla$, $\textrm{div}, \Delta$ and $\Delta _f$ are all time dependent too which makes the analysis more challenging. In the course of the paper, it will become apparent that the super flow inequality

1.15 $$\begin{aligned} \frac{1}{2} \dfrac{\partial g}{\partial t} (x,t) + p u^{p-1}(x,t) {\mathscr {R}ic}^m_f(g)(x,t) \ge - {\textsf k} g(x,t), \end{aligned}$$

relating the metric *g*, the potential *f*, and the positive solution *u* to (1.1) with $p>1$ will play an interesting role. Here we note that

1.16 $$\begin{aligned} {\mathscr {R}ic}_f^m(g)(x,t) = {\mathscr {R}ic} (g)(x,t) + \nabla _{g} \nabla _{g} f (x,t) - [\nabla _{g} f \otimes \nabla _{g} f](x,t)/(m-n), \end{aligned}$$

(and we use the convention described earlier for $m=n$). Interestingly, in this nonlinear slow diffusion context, the super flow inequality (1.15) presents itself as the natural substitute for the super Perelman-Ricci flow inequality that links to linear heat-type equations where $p=1$ (see [36–38, 40]). As will become evident later, the form of this super flow inequality is closely connected with the evolution operator ${{\mathscr {L}}}_p^v$ defined in (1.3). (We refer the reader to Lemma 3.3, Lemmas 7.1 and 7.3 and the entire Section 10 for more discussion on this.)

For the sake of establishing the main results and (local) estimates in this paper we shall make use of the lower bounds

1.17 $$\begin{aligned} \partial _t g (x,t) \ge - 2h g(x,t), \qquad {\mathscr {R}ic}^m_f(g)(x,t) \ge - k g(x,t), \end{aligned}$$

for suitable constants *h*, $k \ge 0$ in a fixed compact space-time cylinder $Q_{R,T}$ with upper base centered at the reference point where the estimate is sought. These bounds on the one hand allow for the use of weighted (Laplace) comparison theorems and on the other provide control on the time derivative of geodesic distances which are two important ingredients in localisation and the proof of gradient estimates. The constants *h*, $k \ge 0$ will appear along with other bounds in the geometry-dependent terms in the ultimate formulation of the local estimates. In the static case, where the metric and potential do not depend on time (i.e., $\partial _t g \equiv 0$ and $\partial _t f \equiv 0$) we can take $h=0$ in (1.17) and our results are immediately seen to cover this special case with the usual assumption of Ricci curvature lower bound (space only) of the type ${\mathscr {R}ic}_f^m(g) \ge - {{\textsf{k}}} g$. It is clear that (1.17) with a local upper bound on the positive solution $u=u(x,t)$ will immediately lead to (1.15) with any choice of constant ${{\textsf{k}}} \ge h + p [\sup u]^{p-1} k \ge 0$, in view of,

1.18 $$\begin{aligned} \partial _t g (x,t) + 2p u^{p-1}(x,t) {\mathscr {R}ic}^m_f(g)(x,t)&\ge -2h g(x,t) - 2k pu^{p-1}(x,t) g(x,t) \nonumber \\&\ge -2 \{ h + kp [\sup u]^{p-1} \} g(x,t) \ge - 2{{\textsf{k}}} g(x,t). \end{aligned}$$

**Plan of the paper.** The main estimates are presented in Theorem 2.1 in Sect. 2 and Theorem 6.1 in Sect. 6 where both estimates are presented in their local forms. These estimates cover different exponent ranges and their proofs rely on different circle of ideas. The global form of these estimates follow by imposing appropriate global bounds on the solution, metric, potential as well as the Bakry-Émery *m*-Ricci curvature tensor and is presented in Theorem 2.4 and Theorem 6.2 respectively. The static case (time independent metrics *g* and potentials *f*) which is an important case is treated as a by-product of the above estimates and is presented in Theorem 2.5 and Theorem 6.3 respectively. As a nice and useful application of these we are able to present parabolic Liouville results which lead to a characterisation of ancient solutions to (1.1). These appear in turn in Theorem 2.6 and Theorem 6.4. As such Sects. 2 and 6 contain the main results of the paper whilst the remaining sections are devoted to developing the necessary apparatus and tools for establishing the results and proofs.

**Notation.** We write $z=z_+ + z_-$ with $z_+=\max (z, 0)$ and $z_-=\min (z, 0)$. Fixing a reference point $x_0 \in \mathscr M$ we denote by $d=d(x,x_0, t)$ the Riemannian distance between *x* and $x_0$ on $\mathscr M$ with respect to $g=g(t)$. We write $\varrho =\varrho (x,x_0,t)$ for the geodesic radial variable measuring distance between *x* and origin $x_0$ at time $t>0$. For a fixed space-time reference point $(x_0,t_0)$ and $R>0$, $T>0$ we define the space-time cylinder

1.19 $$\begin{aligned} Q_{R,T} (x_0,t_0) \equiv \{ (x, t) | d(x, x_0, t) \le R, t_0-T \le t \le t_0 \} \subset \mathscr M \times [t_0-T, t_0]. \end{aligned}$$

When the metric *g* is time independent, we denote by $\mathscr {B}_\varrho (x_0) \subset \mathscr M$ the geodesic ball of radius $\varrho >0$ centred at $x_0$. It is evident that in this case we have

1.20 $$\begin{aligned} Q_{R,T} (x_0,t_0) = \mathscr {B}_R(x_0) \times [t_0-T, t_0] \subset \mathscr M \times [t_0-T, t_0]. \end{aligned}$$

Note that when the choice of the reference point $(x_0, t_0)$ is clear from the context we often abbreviate and write *d*(*x*, *t*), $\varrho (x,t)$ or ${{\mathscr {B}}}_\varrho$, $Q_{R,T}$ respectively.

For a function of multiple variables, we denote its partial derivatives with subscripts accordingly. For instance for $\Gamma =\Gamma (x,u)$ we denote its partial derivatives with respect to $x=(x_1, \dots , x_n)$ or *u* by $\Gamma _x$ or $\Gamma _u$ respectively. Moreover we reserve the notation $\Gamma ^x$ for the function $x \mapsto \Gamma (x, u)$ obtained by freezing the argument *u* and viewing it as a function of *x*. In the sequel we frequently make use of the notations $\nabla \Gamma ^x$ and $\Delta _f \Gamma ^x$.

The Riemann, Ricci and Bakry-Émery *m*-Ricci curvature tensors associated with the metric *g* and potential *f*, in local coordinates $(x_1, \dots , x_n)$, are given by:

1.21 $$\begin{aligned} & {[}\textrm{Riem}(g)]^\ell _{ijk} = \frac{\partial \Gamma ^\ell _{jk}}{\partial x_i} - \frac{\partial \Gamma ^\ell _{ik}}{\partial x_j} + \Gamma ^p_{jk} \Gamma ^\ell _{ip} - \Gamma ^p_{ik} \Gamma ^\ell _{jp}, \end{aligned}$$

1.22 $$\begin{aligned} & {[}{\mathscr {R}ic}(g)]_{ij} = \frac{\partial \Gamma _{ij}^k}{\partial x_k} - \frac{\partial \Gamma _{\ell j}^\ell }{\partial x_i} + \Gamma ^k_{ij} \Gamma ^\ell _{\ell k} - \Gamma ^\ell _{ik} \Gamma ^k_{\ell j}, \end{aligned}$$

1.23 $$\begin{aligned} & {[}{\mathscr {R}ic}_f^m(g)]_{ij} = \frac{\partial \Gamma _{ij}^k}{\partial x_k} - \frac{\partial \Gamma _{\ell j}^\ell }{\partial x_i} + \Gamma ^k_{ij} \Gamma ^\ell _{\ell k} - \Gamma ^\ell _{ik} \Gamma ^k_{\ell j} + \frac{\partial ^2 f}{\partial x_i \partial x_j} - \Gamma ^k_{ij} \frac{\partial f}{\partial x_k} - \frac{1}{m-n} \frac{\partial f}{\partial x_i} \frac{\partial f}{\partial x_j}. \end{aligned}$$

Likewise the *f*-Laplacian (1.2) associated with the metric *g* and potential *f* takes the form:

1.24 $$\begin{aligned} \Delta _f = \underbrace{\frac{1}{\sqrt{|g|}} \frac{\partial }{\partial x_i} \left( \sqrt{|g|} g^{ij} \frac{\partial }{\partial x_j} \right) }_{\Delta } - \underbrace{g^{ij} \frac{\partial f}{\partial x_i} \frac{\partial }{\partial x_j}}_{\langle \nabla f, \cdot \rangle }. \end{aligned}$$

In (1.21)-(1.23) above $\Gamma ^k_{ij} = (g^{k\ell }/2) (\partial g_{j \ell }/\partial x_i + \partial g_{i \ell }/\partial x_j - \partial g_{ij}/\partial x_\ell )$, are the Christoffel symbols of *g* whilst $g_{ij}$, |*g*|, $g^{ij} = (g^{-1})_{ij}$ are the components, determinant and the components of the inverse of *g*.

## A Hamilton-Souplet-Zhang estimate for (1.1): $1< p < 1+1/(\sqrt{2m} +1)$

In this section we present the first set of gradient estimates for the positive solutions to equation (1.1). The estimates as will be seen below are valid in the exponent range $1<p<1+1/(\sqrt{2m} +1)$. Note that $m \ge n$ inherently has the role of dimension for the SMMS. Here the curvature lower bounds are expressed as ${\mathscr {R}ic}_f^m(g) \ge -(m-1)k g$ where the presence of *m* is explicitly felt. Note that *m* is not necessarily an integer.

### Theorem 2.1

Let $(\mathscr M, g, d\mu )$ be a complete smooth metric measure space with $d\mu =e^{-f} dv_g$. Assume the metric and potential are time dependent, of class $\mathscr {C}^2$ and that for suitable constants $k, h \ge 0$ and $m \ge n$ satisfy ${\mathscr {R}ic}_f^m (g) \ge -(m-1)k g$, $\partial _t g \ge -2 h g$ in the space-time cylinder $Q_{R,T}$ with $R, T >0$. Let *u* be a positive solution to (1.1) with $1< p <1+1/(\sqrt{2m} +1)$ and $v =pu^{p-1}/(p-1)$ and $M=\sup _{Q_{R,T}} v$. Then there exists $C=C(p,\beta ,m)>0$ such that for every (*x*, *t*) in $Q_{R/2,T}$ with $t>t_0-T$ we have

2.1 $$\begin{aligned} \frac{|\nabla v|}{v^{\beta /2}}(x,t) \le C \left\{ \begin{array}{ll} \sqrt{h} M^{(1-\beta )/2} + \left[ \dfrac{k^{1/4}}{\sqrt{R}} + \dfrac{1}{R} + \sqrt{k} \right] M^{1-\beta /2} \\ \\ + \dfrac{M^{(1-\beta )/2}}{\sqrt{t-t_0+T}} + \sup _{Q_{R, T}} \left\{ \left[ \dfrac{|\Sigma _x(t,x,v)|}{v^{(3\beta -2)/2}}\right] ^{1/3}\right\} \\ \\ + \sup _{Q_{R, T}}\left\{ v^{(1-\beta )/2} \left[ 2 \Sigma _v(t,x,v)-\dfrac{ \beta \Sigma (t,x,v)}{v} \right] _+^{1/2}\right\} \end{array} \right\} . \end{aligned}$$

Here $\beta \in (\beta _1, \beta _2)$ where $\beta _1<\beta _2< 0$ are the roots of $\beta ^2 + (2-p)/(p -1)\beta +m/2 =0$ and $\Sigma (t,x,v)$ is defined by

2.2 $$\begin{aligned} \Sigma (t,x,v)= p [(p-1)v/p ]^{\frac{p -2}{p-1}} {\mathscr {N}} \left( t,x,[(p-1)v/p]^{\frac{1}{p-1}}\right) . \end{aligned}$$

### Remark 2.2

The particular choice $\beta = -(2-p)/[2(p-1)]$ (easily seen to lie between the roots $\beta _1$, $\beta _2$) leads to the following formulation of the estimate (2.1) that is recorded for future reference:

2.3 $$\begin{aligned} v^{\frac{1}{4}\frac{2-p}{p-1}}|\nabla v| \le C \left\{ \begin{array}{ll} \sqrt{h} M^{\frac{1}{2}+\frac{1}{4}\frac{2-p}{p-1}} + \left[ \dfrac{k^{1/4}}{\sqrt{R}} + \dfrac{1}{R} + \sqrt{k} \right] M^{1+\frac{1}{4}\frac{2-p}{p-1}} \\ \\ + \dfrac{M^{\frac{1}{2}+\frac{1}{4}\frac{2-p}{p-1}}}{\sqrt{t-t_0+T}} + \sup _{Q_{R, T}} \left\{ \left[ v^{\frac{1}{4}\frac{p+2}{p-1}} |\Sigma _x(t,x,v)|\right] ^{1/3}\right\} \\ \\ + \sup _{Q_{R, T}}\left\{ v^\frac{p}{4(p-1)}\left[ 2 \Sigma _v(t,x,v)+\dfrac{ (2-p)\Sigma (t,x,v)}{2(p-1)v} \right] _+^{1/2}\right\} \end{array} \right\} . \end{aligned}$$

### Remark 2.3

We can alternatively rewrite (2.2) as $\Sigma (t,x,v) = p u^{p-2} {{\mathscr {N}}}(t,x,u)$ where *u* and *v* are related via $v =pu^{p-1}/(p-1)$. In passing we point out that the change of variables taking from *u* to *v* is often called the *pressure* transform in literature.

Subject to the bounds in Theorem 2.1 being global in space by passing to the limit $R \rightarrow \infty$ we have the following global (in space) estimate.

### Theorem 2.4

Let $(\mathscr M, g, d\mu )$ be a complete smooth metric measure space with $d\mu =e^{-f} dv_g$. Assume the metric and potential are time dependent, of class $\mathscr {C}^2$ and that for suitable constants $k, h \ge 0$ and $m \ge n$ satisfy ${\mathscr {R}ic}_f^m (g) \ge -(m-1)k g$, $\partial _t g \ge -2 h g$ on $\mathscr M \times [t_0-T, t_0]$. Let *u* be a positive solution to (1.1) with $1< p <1+ 1/(\sqrt{2m} +1)$ and $v =pu^{p-1}/(p-1)$ and $M= \sup v$. Then there exists $C=C(p,\beta ,m)>0$ such that for every $x \in \mathscr M$ and $t_0-T<t \le t_0$ we have

2.4 $$\begin{aligned} \frac{|\nabla v|}{v^{\beta /2}}(x,t) \le C \left\{ \begin{array}{ll} \sqrt{k} M^{1-\beta /2} + \left[ \sqrt{h} + \dfrac{1}{\sqrt{t-t_0+T}} \right] M^{(1-\beta )/2} \\ \\ + \sup _{\mathscr M \times [t_0-T, t_0]} \left\{ \left[ \dfrac{|\Sigma _x(t,x,v)|}{v^{(3\beta -2)/2}}\right] ^{1/3}\right\} \\ \\ + \sup _{\mathscr M \times [t_0-T, t_0]}\left\{ v^{(1-\beta )/2} \left[ 2 \Sigma _v(t,x,v)-\dfrac{ \beta \Sigma (t,x,v)}{v} \right] _+^{1/2}\right\} \end{array} \right\} . \end{aligned}$$

Here $$\beta \in (\beta _1, \beta _2)$$ where $$\beta _1<\beta _2< 0$$ are the roots of $$\beta ^2 + (2-p)/(p -1)\beta$$$$+m/2 =0$$ and $$\Sigma (t,x,v)$$ is as in (2.2).

The special case of time independent metrics and potentials (the static case $\partial _t g \equiv 0$ and $\partial _t f \equiv 0$) is of enough significance to be formulated as a separate corollary. Here we describe the local version. The global version follows by passing to the limit $R\rightarrow \infty$.

### Theorem 2.5

Let $(\mathscr M, g, d\mu )$ be a complete smooth metric measure space with $d\mu =e^{-f} dv_g$ and assume ${\mathscr {R}ic}_f^m (g) \ge -(m-1)k g$ in ${{\mathscr {B}}}_R$ for some $k \ge 0$, $m \ge n$ and $R>0$. Let *u* be a positive solution to (1.1) with $1< p <1+ 1/(\sqrt{2m} +1)$ and $v =pu^{p-1}/(p-1)$ and $M= \sup _{Q_{R,T}} v$. Then there exists $C=C(p,\beta ,m)>0$ such that for every (*x*, *t*) in $Q_{R/2,T}$ with $t>t_0-T$ we have

2.5 $$\begin{aligned} \frac{|\nabla v|}{v^{\beta /2}}(x,t) \le C \left\{ \begin{array}{ll} \left[ \dfrac{k^{1/4}}{\sqrt{R}} + \dfrac{1}{R} + \sqrt{k} \right] M^{1-\beta /2} \\ \\ + \dfrac{M^{(1-\beta )/2}}{\sqrt{t-t_0+T}} + \sup _{Q_{R, T}} \left\{ \left[ \dfrac{|\Sigma _x(t,x,v)|}{v^{(3\beta -2)/2}}\right] ^{1/3}\right\} \\ \\ + \sup _{Q_{R, T}}\left\{ v^{(1-\beta )/2} \left[ 2 \Sigma _v(t,x,v)-\dfrac{ \beta \Sigma (t,x,v)}{v} \right] _+^{1/2}\right\} \end{array} \right\} . \end{aligned}$$

Here $$\beta \in (\beta _1, \beta _2)$$ where $$\beta _1<\beta _2< 0$$ are the roots of $$\beta ^2 + (2-p)/(p -1)\beta$$$$+m/2 =0$$ and $$\Sigma (t,x,v)$$ is as in (2.2).

We end this section with an application of the estimates above to parabolic Liouville-type theorems. Here by an ancient solution $u=u(x,t)$ to (1.1) we mean a solution defined on $\mathscr M$ for all negative times, that is, for all (*x*, *t*) with $x \in \mathscr M$ and $-\infty< t <0$.

### Theorem 2.6

Let $(\mathscr M, g, d\mu )$ be a complete smooth metric measure space with $d\mu =e^{-f}dv_g$ and ${\mathscr {R}ic}^m_f(g) \ge 0$. Assume $[2(2-p)+\beta (p-1)] {{\mathscr {N}}}(u) - 2u{{\mathscr {N}}}_u(u) \ge 0$ for some $\beta \in (\beta _1, \beta _2)$ and all $u>0$ where $1< p <1+ 1/(\sqrt{2m} +1)$. Then any positive ancient solution $u=u(x,t)$ to the nonlinear porous medium equation

2.6 $$\begin{aligned} \partial _t u - \Delta _f u^p = {{\mathscr {N}}}(u(x,t)), \end{aligned}$$

satisfying the growth at infinity

2.7 $$\begin{aligned} u(x,t) = o \left( [\varrho (x) + \sqrt{|t|}]^{2/[(p-1)(2-\beta )]} \right) , \end{aligned}$$

must be spatially constant. In particular, if additionally, ${{\mathscr {N}}}(u) \ge a$ for some $a>0$ and all $u>0$ then (2.6) admits no such ancient solutions.

## Evolution inequalities $\mathbf{I}$: ${\mathscr {L}}_v^p=\partial _t - (p-1)v\Delta _f$ and $w = |\nabla v|^2/v^{\beta }$

In this section we derive evolution identities and inequalities for $w = |\nabla v|^2/v^{\beta }$ where *v* relates to the positive solution *u* through the pressure transform $v =pu^{p-1}/(p-1)$. Here the evolution operator is ${\mathscr {L}}^p_v = \partial _t -(p-1) v \Delta _f$ and $\beta \in {{\mathbb {R}}}$ is to be specified later.

### Lemma 3.1

Let *u* be a positive solution to (1.1) with $p>1$ and let $v =pu^{p-1}/(p-1)$ and $\Sigma =\Sigma (t,x,v)$ be as in (2.2). Then *v* satisfies the evolution equation

3.1 $$\begin{aligned} {\mathscr {L}}_v^p [v] = [\partial _t -(p-1) v \Delta _f] v = |\nabla v|^2 + \Sigma (t,x,v). \end{aligned}$$

### Proof

Using the formulation of *v* and by directly differentiating *u* and $u^p$ we have

3.2 $$\begin{aligned} \partial _t u =&~\partial _t [(p-1)v/p]^{1/(p-1)} = (1/p) [(p-1)v/p]^{\frac{2-p}{p-1}} \partial _t v, \nonumber \\ \nabla u^p =&~\nabla [(p-1)v/p]^{p/(p-1)} = [(p-1)v/p]^{\frac{1}{p-1}} \nabla v, \nonumber \\ \Delta u^p =&~(1/p) [(p-1)v/p]^{\frac{2-p}{p-1}}[(p-1) v \Delta v + |\nabla v|^2]. \end{aligned}$$

Therefore by recalling $\Delta _f u^p = \Delta u^p - \langle \nabla f, \nabla u^p \rangle$ and upon substitution it is seen that

3.3 $$\begin{aligned} \Delta _f u^p&= (1/p) [(p-1)v/p]^{\frac{2-p}{p-1}}[(p-1) v \Delta v + |\nabla v|^2] - [(p-1)v/p]^{\frac{1}{p-1}}\langle \nabla f, \nabla v \rangle \nonumber \\&= (1/p) [(p-1)v/p]^{\frac{2-p}{p-1}}[(p-1) v \Delta _f v + |\nabla v|^2]. \end{aligned}$$

Now referring to equation (1.1) and by substituting the above fragments in the equation we have

3.4 $$\begin{aligned} \square _p u = \partial _t u - \Delta _f u^p&= (1/p) [(p-1)v/p]^{\frac{2-p}{p-1}} (\partial _t v - (p-1)v \Delta _f v - |\nabla v|^2) \nonumber \\&= {\mathscr {N}}\left( t,x,[(p-1)v/p]^{\frac{1}{p-1}}\right) , \end{aligned}$$

which upon a rearrangement of terms and using (2.2) gives the desired conclusion. $\square$

### Lemma 3.2

Let *u* be a positive solution to (1.1) with $p>1$ and set $w = |\nabla v|^2/v^{\beta }$ where $v =pu^{p-1}/(p-1)$ and $\beta \in {{\mathbb {R}}}$. Then *w* satisfies the evolution equation

3.5 $$\begin{aligned} {\mathscr {L}}_v^p [w] =&-2\left[ \frac{1}{2} \partial _t g+(p -1)v{\mathscr {R}ic}_f^m (g) \right] \frac{( \nabla v, \nabla v)}{v^\beta }\\&+\frac{2(p -1)}{v^{\beta }} \left[ |\nabla v|^2 \Delta _f v - v|\nabla \nabla v|^2 -\frac{v \langle \nabla f, \nabla v \rangle ^2}{(m-n)}\right] \nonumber \\&+2[1+\beta (p -1)] \frac{\langle \nabla v, \nabla |\nabla v|^2 \rangle }{v^\beta } -\beta \frac{ |\nabla v|^2 \Sigma (t,x,v)}{v^{\beta +1}}\nonumber \\&-\beta [1+(p -1)(\beta +1)]\frac{|\nabla v|^4}{v^{\beta +1}} +2 \frac{\langle \nabla v, \nabla \Sigma (t,x,v)\rangle }{v^{\beta }}. \nonumber \end{aligned}$$

### Proof

As $w = |\nabla v|^2/v^{\beta }$ and $v =pu^{p-1}/(p-1)$ satisfies (3.1), a direct calculation gives

3.6 $$\begin{aligned} \partial _t w= \partial _t \left[ \frac{|\nabla v|^2}{v^{\beta }}\right] =&~2\frac{\langle \nabla v, \nabla \partial _t v \rangle }{v^\beta } -\frac{[\partial _t g] ( \nabla v, \nabla v)}{v^\beta } - \beta \frac{v^{\beta -1}|\nabla v|^2 \partial _t v}{v^{2\beta }}\nonumber \\ =&~ \frac{2\langle \nabla v, \nabla [(p -1) v \Delta _f v +|\nabla v|^2 +\Sigma (t,x,v)]\rangle }{v^\beta } -\frac{[\partial _t g] ( \nabla v, \nabla v)}{v^\beta } \nonumber \\&-\frac{\beta |\nabla v|^2 [(p -1) v \Delta _f v +|\nabla v|^2 +\Sigma (t,x,v)]}{v^{\beta +1}}\nonumber \\ =&~ 2(p -1)\frac{ |\nabla v|^2 \Delta _f v}{v^\beta } +2(p -1) \frac{\langle \nabla v, \nabla \Delta _f v \rangle }{v^{\beta -1}} +2\frac{\langle \nabla v , \nabla |\nabla v|^2 \rangle }{v^\beta }\nonumber \\&+ 2 \frac{\langle \nabla v, \nabla \Sigma (t,x,v)\rangle }{v^\beta } -\frac{[\partial _t g]( \nabla v, \nabla v)}{v^\beta } - \beta (p -1) \frac{|\nabla v|^2 \Delta _f v}{v^\beta }\nonumber \\&- \beta \frac{|\nabla v|^4}{v^{\beta +1}} - \beta \frac{|\nabla v|^2 \Sigma (t,x,v)}{v^{\beta +1}}. \end{aligned}$$

Furthermore, as $\nabla w = \nabla ( |\nabla v|^2/v^{\beta })= \nabla |\nabla v|^2/v^\beta - \beta |\nabla v|^2 \nabla v/v^{\beta +1}$, by recalling the formulation $\Delta _f w = \Delta w - \langle \nabla f, \nabla w \rangle$ it is seen that

3.7 $$\begin{aligned} \Delta _f w&= {\text{ d }iv} [\nabla |\nabla v|^2/v^\beta - \beta |\nabla v|^2 \nabla v/v^{\beta +1} ]- \langle \nabla f, \nabla |\nabla v|^2/v^\beta - \beta |\nabla v|^2 \nabla v/v^{\beta +1} \rangle \nonumber \\&=\frac{\Delta _f |\nabla v|^2}{v^\beta } - 2 \beta \frac{\langle \nabla v, \nabla |\nabla v|^2 \rangle }{v^{\beta +1}} -\beta \frac{|\nabla v|^2 \Delta _f v}{v^{\beta +1}} +\beta (\beta +1) \frac{ |\nabla v|^4}{v^{\beta +2}}. \end{aligned}$$

Putting (3.6)-(3.7) together and rearranging terms results in

3.8 $$\begin{aligned} {[}\partial _t-(p-1) v\Delta _f] w =&~2(p -1) \frac{|\nabla v|^2 \Delta _f v}{v^{\beta }} +2[1+ \beta (p -1)] \frac{\langle \nabla v, \nabla |\nabla v|^2 \rangle }{v^\beta }\nonumber \\&+2(p -1) \frac{\langle \nabla v, \nabla \Delta _f v \rangle }{v^{\beta -1}} -(p -1) \frac{\Delta _f |\nabla v|^2}{v^{\beta -1}}\nonumber \\&-[\beta +(p -1) \beta (\beta +1)]\frac{|\nabla v|^4}{v^{\beta +1}} -\frac{[\partial _t g] ( \nabla v, \nabla v)}{v^\beta }\nonumber \\&+2 \frac{\langle \nabla v, \nabla \Sigma (t,x,v) \rangle }{v^{\beta }} - \beta \frac{ |\nabla v|^2 \Sigma (t,x,v)}{v^{\beta +1}}. \end{aligned}$$

Applying the weighted Bochner-Weitzenböck formula (1.8) to the right-hand side gives

3.9 $$\begin{aligned} {[}\partial _t-(p-1) v\Delta _f] w =&-\frac{2(p -1)}{v^{\beta -1}}\left[ |\nabla \nabla v|^2 +{\mathscr {R}ic}_f^m (\nabla v, \nabla v) +\frac{\langle \nabla f, \nabla v \rangle ^2}{(m-n)}\right] \nonumber \\&+ 2(p -1) \frac{|\nabla v|^2 \Delta _f v}{v^{\beta }} + 2[1+\beta (p -1)] \frac{\langle \nabla v, \nabla |\nabla v|^2 \rangle }{v^\beta }\nonumber \\&- [\beta +(p -1) \beta (\beta +1)]\frac{|\nabla v|^4}{v^{\beta +1}} - \frac{[\partial _t g] ( \nabla v, \nabla v)}{v^\beta }\nonumber \\&+ 2 \frac{\langle \nabla v, \nabla \Sigma (t,x,v)\rangle }{v^{\beta }} - \beta \frac{|\nabla v|^2 \Sigma (t,x,v)}{v^{\beta +1}}, \end{aligned}$$

which after rearranging term leads to the desired identity. $\square$

### Lemma 3.3

Under the assumptions of Lemma 3.2, if the metric $g$ and potential $f$ satisfy the super flow inequality

3.10 $$\begin{aligned} \frac{1}{2} \partial _t g + (p-1) v {\mathscr {R}ic}_f^m (g) \ge - {{\textsf{k}}} g, \end{aligned}$$

then *w* satisfies the evolution inequality

3.11 $$\begin{aligned} {\mathscr {L}}_v^p [w] \le&~ 2 {{\textsf{k}}} w + 2 [1+\beta (p -1)] \langle \nabla v , \nabla w \rangle \nonumber \\&+ ( p -1) \left[ \beta ^2 -\frac{p -2}{p -1} \beta +\frac{m}{2} \right] v^{\beta -1} w^2 \nonumber \\&+ 2 \frac{\langle \nabla v, \Sigma _x(t,x,v) \rangle }{v^\beta } +\left[ 2 \Sigma _v(t,x,v) -\frac{ \beta \Sigma (t,x,v)}{v} \right] w. \end{aligned}$$

Note that k here may be a constant or more generally a function of space and time.    

### Proof

Starting from (3.5) and noting $\langle \nabla v, \nabla w \rangle = \langle \nabla v, \nabla |\nabla v|^2 \rangle /v^\beta -\beta |\nabla v|^4/v^{\beta +1}$ we can write upon substitution

3.12 $$\begin{aligned} {[}\partial _t-(p-1) v\Delta _f] w =&-2\left[ \frac{1}{2} \partial _t g+(p -1)v{\mathscr {R}ic}_f^m(g)\right] \frac{( \nabla v, \nabla v)}{v^\beta }\nonumber \\&+\frac{2(p -1)}{v^{\beta }} \left[ |\nabla v|^2 \Delta _f v - v|\nabla \nabla v|^2 -\frac{v \langle \nabla f, \nabla v \rangle ^2}{(m-n)}\right] \nonumber \\&+2[1+\beta (p -1)] \langle \nabla v, \nabla w \rangle -\beta \frac{ |\nabla v|^2 \Sigma (t,x,v)}{v^{\beta +1}}\nonumber \\&+[(p-1)\beta ^2-(p-2)\beta ]\frac{|\nabla v|^4}{v^{\beta +1}} +2 \frac{\langle \nabla v, \nabla \Sigma (t,x,v)\rangle }{v^{\beta }}. \end{aligned}$$

Now by writing $\langle \nabla v, \nabla \Sigma (t,x,v) \rangle =\langle \nabla v, \Sigma _x(t,x,v) \rangle + \Sigma _v(t,x,v) |\nabla v|^2$ and substituting this back in the equation it follows that

3.13 $$\begin{aligned} {[}\partial _t-(p-1) v\Delta _f] w =&-2\left[ \frac{1}{2}\partial _t g+(p -1)v{\mathscr {R}ic}_f^m(g)\right] \frac{( \nabla v, \nabla v)}{v^\beta }\nonumber \\&+\frac{2(p -1)}{v^{\beta }} \left[ |\nabla v|^2 \Delta _f v - v|\nabla \nabla v|^2 -\frac{v \langle \nabla f, \nabla v \rangle ^2}{(m-n)}\right] \nonumber \\&+2[1+\beta (p -1)] \langle \nabla v, \nabla w \rangle +\left[ 2 \Sigma _v(t,x,v) -\beta \frac{ \Sigma (t,x,v)}{v}\right] \frac{|\nabla v|^2}{v^{\beta }}\nonumber \\&+[(p-1)\beta ^2-(p-2)\beta ]\frac{|\nabla v|^4}{v^{\beta +1}} +2\frac{\langle \nabla v, \Sigma _x(t,x,v) \rangle }{v^\beta }. \end{aligned}$$

Next referring to the second line in the above and by making note of the inequality

3.14 $$\begin{aligned} |\nabla \nabla v|^2 + \frac{\langle \nabla f, \nabla v \rangle ^2}{m-n} \ge \frac{(\Delta v)^2}{n} + \frac{\langle \nabla f, \nabla v \rangle ^2}{m-n} \ge \frac{(\Delta _f v)^2}{m}, \end{aligned}$$

we can write

3.15 $$\begin{aligned} \frac{2(p -1)}{v^{\beta }}&\left[ |\nabla v|^2 \Delta _f v -v|\nabla \nabla v|^2 - \frac{v\langle \nabla f, \nabla v \rangle ^2}{m-n}\right] \nonumber \\&\le 2(p -1) \frac{1}{v^{\beta -1}} \left[ \frac{|\nabla v|^2 \Delta _f v}{v}- \frac{(\Delta _f v)^2}{m}\right] \nonumber \\&\le 2(p -1) \frac{1}{v^{\beta -1}} \left[ -\left( \frac{\sqrt{m}}{2} \frac{|\nabla v|^2}{v} - \frac{\Delta _f v}{\sqrt{m}} \right) ^2 +\frac{m}{4} \frac{|\nabla v|^4}{v^2} \right] \le \frac{m(p -1)}{2} \frac{|\nabla v|^4}{v^{\beta +1}}. \end{aligned}$$

Therefore substituting the above inequality in (3.13) results in

3.16 $$\begin{aligned} {{\mathscr {L}}}_v^p[w] \le&-2\left[ \frac{1}{2}\partial _t g+(p -1)v{\mathscr {R}ic}_f^m (g)\right] \frac{( \nabla v, \nabla v)}{v^\beta } +2[1+ \beta (p -1)] \langle \nabla v, \nabla w \rangle \nonumber \\&+ \left[ \frac{m}{2} (p -1)+ (p-1)\beta ^2-(p-2)\beta \right] \frac{|\nabla v|^4}{v^{\beta +1}} \nonumber \\&+2\frac{\langle \nabla v, \Sigma _x(t,x,v) \rangle }{v^\beta } +\left[ 2 \Sigma _v(t,x,v) -\beta \frac{ \Sigma (t,x,v)}{v}\right] \frac{|\nabla v|^2}{v^{\beta }}. \end{aligned}$$

The conclusion now follows by making use of the super flow (3.10), making the substitution $w = |\nabla v|^2/v^{\beta }$ and rearranging terms. $\square$

## Space-time cut-offs, cylindrical localisation and the proof of Theorem 2.1

We start this section with a construction of smooth space-time cut-off functions that will be used in the proof of Theorem 2.1. First, let us fix a reference point $(x_0,t_0)$ with $x_0 \in \mathscr M$, $t_0 \in {{\mathbb {R}}}$, and pick $R, T>0$ and then $\tau \in (t_0-T, t_0]$. The following standard lemma grants the existence of a smooth *profile* function $\bar{\eta }={\bar{\eta }}(\varrho , t)$ with variables $\varrho \ge 0$ and $t_0-T \le t \le t_0$ that satisfies a suitable set of properties and bounds.

### Lemma 4.1

Fix $t_0 \in {{\mathbb {R}}}$ and let $R, T>0$. Given $\tau \in (t_0-T, t_0]$ there exists a smooth function $\bar{\eta }:[0,\infty ) \times [t_0-T, t_0] \rightarrow \mathbb {R}$ such that the following properties hold:

(i) $$\textrm{supp} \, \bar{\eta }(\varrho ,t) \subset [0,R] \times [t_0-T, t_0]$$ and $$0 \le \bar{\eta }(\varrho ,t) \le 1$$ in $$[0,R] \times [t_0-T, t_0]$$,
(ii) $\bar{\eta }=1$ in $[0,R/2] \times [\tau , t_0]$ and $\partial \bar{\eta }/\partial \varrho =0$ in $[0,R/2] \times [t_0-T, t_0]$, respectively,
(iii) there exists $c>0$ such that
  4.1 $$\begin{aligned} \frac{|\partial _t \bar{\eta }|}{\sqrt{{\bar{\eta }}}} \le \frac{c}{\tau -t_0+T}, \end{aligned}$$
  in $[0,\infty )\times [t_0-T,t_0]$ and $\bar{\eta }(\varrho ,t_0-T)=0$ for all $\varrho \in [0,\infty )$.
(iv) $-c_a \bar{\eta }^a/R \le \partial _\varrho \bar{\eta } \le 0$ and $|\partial _{\varrho \varrho } \bar{\eta }| \le c_a \bar{\eta }^a / R^2$ hold on $[0, \infty )\times [t_0-T, t_0]$ for every $0<a<1$ and some $c_a>0$.

Using this lemma we next introduce a smooth space-time cut-off function $\eta =\eta (x,t)$ by way of composition of the profile $\bar{\eta }$ with the geodesic radial variable $\varrho$, namely,

4.2 $$\begin{aligned} \eta (x,t) = \bar{\eta }(\varrho (x,t), t), \qquad (x, t) \in \mathscr M \times [t_0-T, t_0]. \end{aligned}$$

It is clear that $0 \le \eta \le 1$ and $\textrm{supp}\,\eta \subset Q_{R,T} = \{(x,t) | d(x,x_0, t) \le R, t_0-T \le t \le t_0\}$ whilst $\eta \equiv 1$ on $\{(x,t) | d(x,x_0, t) \le R/2, \tau \le t \le t_0\}$. Moreover, referring to (4.2) a straightforward calculation gives

- $$\nabla \eta = \partial _\varrho {\bar{\eta }} \nabla \varrho $$,
- $$\partial _t \eta = \partial _\varrho {\bar{\eta }} \partial _t \varrho + \partial _t {\bar{\eta }}$$,
- $$\Delta _f \eta = \partial _{\varrho \varrho } {\bar{\eta }} |\nabla \varrho |^2 + \partial _\varrho {\bar{\eta }} \Delta _f \varrho $$.

As a result it is easily seen that

4.3 $$\begin{aligned} {\mathscr {L}}_v^p [\eta ]&= [\partial _t - (p -1)v \Delta _f] \eta \nonumber \\&= \partial _\varrho {\bar{\eta }} \partial _t \varrho + \partial _t {\bar{\eta }} - (p-1) v [\partial _{\varrho \varrho } {\bar{\eta }} |\nabla \varrho |^2 + \partial _\varrho {\bar{\eta }} \Delta _f \varrho ] \nonumber \\&= \partial _\varrho {\bar{\eta }} {\mathscr {L}}_v^p [\varrho ] + \partial _t {\bar{\eta }} - (p-1) v \partial _{\varrho \varrho } {\bar{\eta }} |\nabla \varrho |^2. \end{aligned}$$

Another auxiliary result on ${\mathscr {L}}_v^p$ that will be needed later on is described below.

### Lemma 4.2

For functions $u=u(x,t)$ and $w=w(x,t)$ of class ${\mathscr {C}}^2$ we have

4.4 $$\begin{aligned} u{\mathscr {L}}_v^p[uw] = uw{\mathscr {L}}_v^p[u] - 2(p -1)v [\langle \nabla u, \nabla (u w) \rangle -|\nabla u|^2 w]+u^2{\mathscr {L}}_v^p [w]. \end{aligned}$$

### Proof

Using $\partial _t (uw) = w \partial _t u + u \partial _t w$ and $\Delta _f (uw) = w \Delta _f u + 2 \langle \nabla u, \nabla w \rangle + u \Delta _f w$ we can write by substitution

4.5 $$\begin{aligned} {\mathscr {L}}_v^p [u w]&= [\partial _t-(p -1)v \Delta _f] (u w) \nonumber \\&= w \partial _t u + u \partial _t w - (p-1) v [w \Delta _f u + 2 \langle \nabla u, \nabla w \rangle + u \Delta _f w] \nonumber \\&= w [\partial _t-(p -1)v \Delta _f] u-2(p-1)v\langle \nabla u, \nabla w \rangle +u[\partial _t-(p -1)v \Delta _f] w. \end{aligned}$$

Now multiplying through by *u* and using $u \langle \nabla u, \nabla w \rangle = \langle \nabla u, \nabla (uw) \rangle - w |\nabla u|^2$ gives

4.6 $$\begin{aligned} u{\mathscr {L}}_v^p [u w] =&~ uw [\partial _t-(p -1)v \Delta _f] u - 2(p -1)v [\langle \nabla u ,\nabla (u w) \rangle - |\nabla u|^2 w] \nonumber \\&+u^2[\partial _t-(p -1)v \Delta _f] w, \end{aligned}$$

which is the desired identity. $\square$

### Proof of Theorem 2.1

We begin by considering the localised function $\eta w$ where $\eta$ is the cut-off function from (4.2). An application of Lemma 4.2 then gives

4.7 $$\begin{aligned} {\mathscr {L}}_v^p[\eta w] = w{\mathscr {L}}_v^p[\eta ] - 2(p -1)v [\langle \nabla \eta ,\nabla (\eta w) \rangle - |\nabla \eta |^2 w]/\eta +\eta {\mathscr {L}}_v^p [w]. \end{aligned}$$

We now wish to utilise the bound on ${\mathscr {L}}_v^p[w]$ from (3.11) in Lemma 3.3. Towards this end invoking the assumptions in Theorem 2.1 we have the super flow inequality (3.10) with ${{\textsf{k}}} = (p-1)(m-1) k v + h$ [we can also take ${{\textsf{k}}} = (p-1)(m-1) k M + h$ as $0<v \le M$]. Therefore substituting this together with $\eta \langle \nabla v, \nabla w \rangle = \langle \nabla v, \nabla (\eta w) \rangle - w \langle \nabla v, \nabla \eta \rangle$ then leads to

4.8 $$\begin{aligned} {\mathscr {L}}_v^p [\eta w] \le&~ w {\mathscr {L}}_v^p [\eta ] -2(p -1) v \left\langle \frac{\nabla \eta }{\eta } , \nabla (\eta w) \right\rangle + 2(p -1)v \frac{|\nabla \eta |^2}{\eta } w \nonumber \\&+ 2[(p - 1) (m-1)k v + h] \eta w +2 [1+\beta (p -1)] \langle \nabla v , \nabla (\eta w) \rangle \nonumber \\&-2 [1+\beta (p -1)] w\langle \nabla v , \nabla \eta \rangle + ( p -1) \left[ \beta ^2 -\frac{p -2}{p -1} \beta +\frac{m}{2} \right] v^{\beta -1} \eta w^2\nonumber \\&+ 2\eta \frac{\langle \nabla v, \Sigma _x(t,x,v) \rangle }{v^\beta } + \left[ 2 \Sigma _v(t,x,v) -\frac{ \beta \Sigma (t,x,v)}{v} \right] \eta w. \end{aligned}$$

Assume now that the localised function $\eta w$ is maximised at the point $(x_1, t_1)$ in the compact set $\{d(x,x_0, t) \le R, t_0-T \le t \le \tau \} \subset \mathscr M \times [t_0-T, t_0]$. Additionally, we can assume that $(\eta w)(x_1, t_1) >0$ as otherwise the conclusion of the theorem is true with $w(x, \tau ) \le 0$ for all $d(x, x_0, \tau ) \le R/2$. In particular $t_1>t_0-T$ and at the point $(x_1,t_1)$ we have $\Delta _f(\eta w) \le 0$, $\partial _t (\eta w) \ge 0$ and $\nabla (\eta w) =0$. Thus at the point $(x_1,t_1)$ we also have ${\mathscr {L}}_v^p [\eta w] \ge 0$. From (4.8) upon rearranging of terms it therefore follows that

4.9 $$\begin{aligned} - ( p -1) \left[ \beta ^2 -\frac{p -2}{p -1} \beta +\frac{m}{2} \right] v^{\beta -1} \eta w^2 \le&~ 2[(p - 1)(m-1) k v + h] \eta w\nonumber \\&- 2 [1+\beta (p -1)] w\langle \nabla v , \nabla \eta \rangle \nonumber \\&+ 2(p -1)v \frac{|\nabla \eta |^2}{\eta } w + w {\mathscr {L}}_v^p [\eta ]\nonumber \\&+\left[ 2 \Sigma _v(t,x,v)-\frac{ \beta \Sigma (t,x,v)}{v} \right] \eta w\nonumber \\&+ 2\eta \frac{\langle \nabla v, \Sigma _x(t,x,v) \rangle }{v^\beta }. \end{aligned}$$

Now it is easily seen that the quadratic expression $\beta ^2 -(p -2)/(p -1) \beta +m/2$ has the discriminant $[(p -2)/(p -1)]^2 -2m$ which is positive only when $p \in (1,1+1/[\sqrt{2m}+1])$. Thus denoting the roots of this quadratic expression by $\beta _1<\beta _2< 0$, it is evident upon choosing $\beta \in (\beta _1, \beta _2)$ that this quadratic expression will be negative. With this choice of $\beta$ we now set $-(p -1)[\beta ^2 -(p -2)/(p -1) \beta +m/2] =2/ \gamma$ where $\gamma > 0$. Therefore returning to (4.9) and multiplying through by $\gamma v^{1-\beta }$ we can write

4.10 $$\begin{aligned} 2 \eta w^2 \le&~ 2 \gamma [(p - 1)(m-1) k v^{2-\beta } + h v^{1-\beta }]\eta w\nonumber \\&- 2 \gamma [1+\beta (p -1)] v^{1-\beta }w\langle \nabla v , \nabla \eta \rangle \nonumber \\&+ 2 \gamma (p -1) v^{2-\beta } \frac{|\nabla \eta |^2}{\eta } w + \gamma v^{1-\beta } w {\mathscr {L}}_v^p [\eta ]\nonumber \\&+\left[ 2 \Sigma _v(t,x,v)-\frac{ \beta \Sigma (t,x,v)}{v} \right] \gamma v^{1-\beta }\eta w\nonumber \\&+ 2 \gamma v^{1-\beta } \eta \frac{\langle \nabla v, \Sigma _x(t,x,v) \rangle }{v^\beta }. \end{aligned}$$

Now we proceed onto bounding the expression on the right-hand side of (4.10). Towards this end, starting from the first line, upon recalling $0\le \eta \le 1$ we can write

4.11 $$\begin{aligned} 2 \gamma (p - 1)(m-1) k v^{2-\beta } \eta w&\le 2 \gamma (p - 1)(m-1) k v^{2-\beta } \eta ^{1/2} w\nonumber \\&\le \frac{1}{7} \eta w^2 + C \gamma ^2 (p-1)^2 (m-1)^2 k^2(\sup _{Q_{R, T}} v)^{4-2\beta }, \end{aligned}$$

and

4.12 $$\begin{aligned} 2 \gamma h v^{1-\beta } \eta w&\le 2 \gamma h v^{1-\beta } \eta ^{1/2} w \le \frac{1}{7} \eta w^2 + C \gamma ^2 h^2 (\sup _{Q_{R, T}} v)^{2-2\beta }. \end{aligned}$$

Note that in view of $\beta <0$ we have $2- \beta >0$ and $1- \beta >0$. Moving to the second line, by recalling $w = |\nabla v|^2/v^{\beta }$ and using (*iv*) in Lemma 4.1, we have

4.13 $$\begin{aligned} -2 \gamma [1+\beta (p -1)] v^{1-\beta }w \langle \nabla v , \nabla \eta \rangle&\le 2 \gamma |1+\beta (p -1)| v^{1-\beta }w |\nabla v| |\nabla \eta |\nonumber \\&\le 2 \gamma |1+\beta (p -1)| v^{1-\beta /2} \eta ^{3/4} w^{3/2} \frac{|\nabla \eta |}{\eta ^{3/4}}\nonumber \\&\le \frac{1}{7} (\eta ^{3/4} w^{3/2})^{4/3} + C \gamma ^4 [1+\beta (p -1)]^4 \left[ \frac{|\nabla \eta |}{\eta ^{3/4}}\right] ^4 v^{4-2\beta }\nonumber \\&\le \frac{1}{7} \eta w^2 + C \gamma ^4\frac{[1+\beta (p -1)]^4}{R^4} (\sup _{Q_{R, T}} v)^{4-2\beta }. \end{aligned}$$

In much the same way for the next term by taking advantage of (*iv*) in Lemma 4.1 we can write

4.14 $$\begin{aligned} 2 \gamma (p -1) v^{2-\beta } \frac{|\nabla \eta |^2}{\eta } w&= 2\gamma (p -1) v^{2-\beta } \frac{|\nabla \eta |^2}{\eta ^{3/2}} \eta ^{1/2} w \nonumber \\&\le \frac{1}{7} \eta w^2 +C \gamma ^2 (p-1)^2 \left[ \frac{|\nabla \eta |}{\eta ^{3/4}} \right] ^4 v^{4-2\beta }\nonumber \\&\le \frac{1}{7} \eta w^2 + C \gamma ^2\frac{(p -1)^2}{R^4}(\sup _{Q_{R, T}} v)^{4-2\beta }. \end{aligned}$$

We now come to the term $\gamma v^{1-\beta } w {\mathscr {L}}_v^p [\eta ] = \gamma v^{1-\beta } w[\partial _t - (p -1)v \Delta _f] \eta$. To handle this term we consider the space and time derivatives separately and provide a lower bound for each.

- Bounding $\mathbf{I}=\gamma (p-1) v^{2-\beta } w (-\Delta _f \eta )$: By virtue of ${\mathscr {R}ic}_f^m(g) \ge - (m-1)k g$ we have $\Delta _f \varrho \le (m-1) \sqrt{k}\coth (\sqrt{k} \varrho )$ and so from (4.2), $\eta$ being radial and ${\bar{\eta }}_\varrho \le 0$, it follows that
  4.15 $$\begin{aligned} \Delta _f \eta = {\bar{\eta }}_{\varrho \varrho }|\nabla \varrho |^2 + {\bar{\eta }}_\varrho \Delta _f \varrho \ge {\bar{\eta }}_{\varrho \varrho } + {\bar{\eta }}_\varrho (m-1)\sqrt{k} \coth (\sqrt{k} \varrho ). \end{aligned}$$
  Now using the bound $\sqrt{k} \coth (\sqrt{k} \varrho )\le \sqrt{k} \coth (\sqrt{k} R/2) \le (2+\sqrt{k} R)/R$ for $0 \le \varrho \le R/2$ and noting that ${\bar{\eta }}_\varrho = 0$ for $0 \le \varrho \le R/2$, it follows that
  4.16 $$\begin{aligned} -\Delta _f \eta&\le - [ {\bar{\eta }}_{\varrho \varrho } + {\bar{\eta }}_\varrho (m-1)\sqrt{k} \coth (\sqrt{k} R/2)]\nonumber \\&\le |{\bar{\eta }}_{\varrho \varrho }| + (m-1)\left( \frac{2}{R} +\sqrt{k}\right) |{\bar{\eta }}_\varrho |. \end{aligned}$$
  Therefore using Young’s inequality and (*iv*) in Lemma 4.1 we can write
  4.17 $$\begin{aligned}&\gamma (p -1) v^{2-\beta } w (-\Delta _f \eta )\\&\le \gamma (p -1) v^{2-\beta } w \left[ |{\bar{\eta }}_{\varrho \varrho }| +(m-1)\left( \frac{2}{R} +\sqrt{k} \right) |{\bar{\eta }}_\varrho | \right] \nonumber \\&\le \gamma (p -1) v^{2-\beta } \sqrt{\eta }w \left[ \frac{|{\bar{\eta }}_{\varrho \varrho }|}{\sqrt{\eta }} +(m-1)\left( \frac{2}{R} +\sqrt{k} \right) \frac{|{\bar{\eta }}_\varrho |}{\sqrt{\eta }} \right] \nonumber \\&\le \frac{1}{14} \eta w^2 + C\gamma ^2 (p -1)^2 \left[ \left( \frac{|{\bar{\eta }}_{\varrho \varrho }|}{\sqrt{\eta }}\right) ^2 +(m-1)^2\left( \frac{1}{R^2} +k \right) \left( \frac{|{\bar{\eta }}_\varrho |}{\sqrt{\eta }}\right) ^2 \right] v^{4-2\beta }\nonumber \\&\le \frac{1}{14} \eta w^2 + C \gamma ^2(p-1)^2(m -1)^2 \left[ \frac{1+ k R^2}{R^4}\right] (\sup _{Q_{R, T}} v)^{4-2\beta }. \end{aligned}$$
- Bounding $\mathbf{II}=\gamma v^{1-\beta }w \partial _t \eta$: We use Lemma 4.1 and $\partial _t \varrho (x,t) \ge - h R$ in $Q_{R,T}$. Let us first justify the latter inequality. Fix *x* and *t* such that $d(x, x_0, t)<R$. Let $X(x_0,x)$ be the set of all minimal geodesics $\lambda =\lambda (s): [0, 1] \rightarrow \mathscr M$ with respect to *g*(*t*) connecting the reference point $x_0 =\lambda (0)$ to $x=\lambda (1)$ and let $\Omega (x_0,x)$ be the set of all ${{\mathscr {C}}}^1$ curves connecting $x_0$ to *x*. By using Lemma B.40 p. 531 in [13] we can write
  4.18 $$\begin{aligned} \partial _t \varrho (x,t)&= \frac{\partial }{\partial t} d (x, x_0; t) = \frac{\partial }{\partial t} \left\{ \inf _{\omega \in \Omega (x_0, x)} \int _0^1 |\omega '(s)|_{g(t)} \, ds \right\} \nonumber \\&= \frac{\partial }{\partial t} \left\{ \inf _{\omega \in \Omega (x_0, x)} \int _0^1 \sqrt{[g(t)] (\omega '(s), \omega '(s))} \, ds \right\} \nonumber \\&= \inf _{\lambda \in X(x_0,x)} \int _0^1 \frac{[\partial _t g(t)](\lambda '(s), \lambda '(s))}{2 \sqrt{[g(t)] (\lambda '(s), \lambda '(s))}} \, ds \nonumber \\&= \inf _{\lambda \in X(x_0,x)} \int _0^1 \frac{[\partial _t g(t)](\lambda '(s), \lambda '(s))}{2|\lambda '(s)|_{g(t)}} \, ds. \end{aligned}$$
  Hence making note of the lower bound $\partial _t g \ge -2h g$ in $Q_{R,T}$ with $h \ge 0$ gives
  4.19 $$\begin{aligned} \partial _t \varrho (x,t)&\ge \inf _{\lambda \in X(x_0,x)} \int _0^1 - h |\lambda ' (s)|_{g(t)} \, ds = \inf _{\lambda \in X(x_0,x)} \left[ - h \underbrace{\int _0^1 |\lambda ' (s)|_{g(t)} \, ds}_{=\varrho (x,t)} \right] \nonumber \\&\ge - h \varrho (x, t) \ge - h R, \end{aligned}$$
  which is the desired inequality. Next, upon noting $\partial _t \eta = \bar{\eta }_t + {\bar{\eta }}_\varrho \partial _t \varrho$ and (*iii*) in Lemma 4.1 we have
  4.20 $$\begin{aligned} \partial _t \eta&= \bar{\eta }_t + {\bar{\eta }}_\varrho \partial _t \varrho \le |{\bar{\eta }}_t| - h R {\bar{\eta }}_\varrho \nonumber \\&\le |{\bar{\eta }}_t| + h R |{\bar{\eta }}_\varrho | \le C \left[ \frac{1}{\tau -t_0+T} + h \right] \sqrt{\eta }. \nonumber \end{aligned}$$
  Hence we can write
  4.21 $$\begin{aligned} \gamma v^{1-\beta }w \partial _t \eta&= \gamma \sqrt{\eta }v^{1-\beta } w \frac{\partial _t \eta }{\sqrt{\eta }} \le C \gamma \sqrt{\eta }v^{1-\beta } w \left[ \frac{1}{\tau -t_0+T}+h \right] \nonumber \\&\le \frac{1}{14} \eta w^2 + C \gamma ^2 \left[ \frac{1}{(\tau -t_0+T)^2} + h^2\right] (\sup _{Q_{R, T}} v)^{2-2\beta }. \end{aligned}$$

By putting together the bounds on the fragments $\mathbf{I}$ and $\mathbf{II}$ from above we arrive at

4.22 $$\begin{aligned} \gamma v^{1-\beta } w {\mathscr {L}}_v^p [\eta ] =&~\overbrace{\gamma (p-1) v^{2-\beta } w (-\Delta _f) \eta }^{\mathbf{I}} + \overbrace{\gamma v^{1-\beta } w\partial _t \eta }^{\mathbf{II}} \nonumber \\ \le&~\frac{1}{7} \eta w^2 + C \gamma ^2 \left[ \frac{1}{(\tau -t_0+T)^2} + h^2\right] (\sup _{Q_{R, T}} v)^{2-2\beta }\nonumber \\&+ C \gamma ^2(p -1)^2 (m-1)^2\left[ \frac{1+ k R^2}{R^4}\right] (\sup _{Q_{R, T}} v)^{4-2\beta }. \end{aligned}$$

Finally for the two remaining terms involving the nonlinearity $\Sigma (t,x,v)$, first upon recalling $0\le \eta \le 1$ and utilising Young’s inequality we have

4.23 $$\begin{aligned} \gamma v^{1-\beta }\eta w \bigg [2 \Sigma _v(t,x,v)&-\frac{ \beta \Sigma (t,x,v)}{v} \bigg ] \le \gamma v^{1-\beta } \sqrt{\eta }w \left[ 2 \Sigma _v(t,x,v)-\frac{ \beta \Sigma (t,x,v)}{v} \right] _+ \nonumber \\&\le \frac{1}{7} \eta w^2 + C \gamma ^2 v^{2(1-\beta )} \left[ 2 \Sigma _v(t,x,v)-\frac{ \beta \Sigma (t,x,v)}{v} \right] _+^2 \nonumber \\&\le \frac{1}{7} \eta w^2 + C \gamma ^2 \sup _{Q_{R, T}} \left\{ v^{2(1-\beta )}\left[ 2 \Sigma _v(t,x,v)-\frac{ \beta \Sigma (t,x,v)}{v} \right] _+^2\right\} . \end{aligned}$$

Likewise, by using the Cauchy-Schwarz and Young inequalities respectively we can bound the last term in (4.10) as

4.24 $$\begin{aligned} 2 \gamma v^{1-\beta } \eta \frac{\langle \nabla v, \Sigma _x(t,x,v) \rangle }{v^\beta } \le&~2 \gamma v^{1-\beta } \eta |\nabla v| \frac{|\Sigma _x(t,x,v)|}{v^\beta } \nonumber \\ \le&~ 2 \gamma \eta ^{1/4} w^{1/2} v^{1- \beta /2} \frac{|\Sigma _x(t,x,v)|}{v^\beta } \nonumber \\ \le&~\frac{1}{7} \eta w^2 + C \gamma ^{4/3} \left[ \frac{|\Sigma _x(t,x,v)|}{v^{(3\beta -2)/2}} \right] ^{4/3} \nonumber \\ \le&~ \frac{1}{7} \eta w^2+C \gamma ^{4/3} \sup _{Q_{R, T}} \left\{ \left[ \frac{|\Sigma _x(t,x,v)|}{v^{(3\beta -2)/2}} \right] ^{4/3}\right\} . \end{aligned}$$

Completing the estimate of the individual terms on the right-hand side of (4.10), by inserting these estimates back into the inequality and after basic considerations and adjusting constants, we arrive at the following bound at the space-time point $(x_1, t_1)$:

4.25 $$\begin{aligned} \eta w^2 \le&~C\gamma ^2 \left\{ \begin{array}{ll} (p -1)^2 (m-1)^2\left[ \dfrac{1+ k R^2}{R^4}\right] +\dfrac{(p -1)^2}{R^4} \\ \\ +\dfrac{\gamma ^2 [1+\beta (p -1)]^4}{R^4} +(p-1)^2 (m-1)^2 k^2 \end{array} \right\} (\sup _{Q_{R, T}} v)^{4-2\beta } \nonumber \\&+C \left\{ \begin{array}{ll} \gamma ^{2} \left[ 2h^2+\dfrac{1}{(\tau -t_0+T)^2}\right] (\sup _{Q_{R, T}} v)^{2-2\beta } \\ \\ +\gamma ^{4/3}\sup _{Q_{R, T}} \left\{ \left[ \dfrac{|\Sigma _x(t,x,v)|}{v^{(3\beta -2)/2}}\right] ^{4/3}\right\} \\ \\ + \gamma ^{2} \sup _{Q_{R, T}} \left\{ v^{2(1-\beta )}\left[ 2 \Sigma _v(t,x,v)-\dfrac{ \beta \Sigma (t,x,v)}{v} \right] _+^2\right\} \end{array} \right\} . \end{aligned}$$

Recalling now that $M=\sup _{Q_{R,T}} v$, and as and a result of $\beta \in (\beta _1, \beta _2)$ that $2-2\beta \ge 2$ and $4-2\beta \ge 4$, it follows from (4.25) upon absorbing $\gamma$ into *C* and adjusting the constant if necessary that

4.26 $$\begin{aligned} \eta w^2 \le C(\gamma )&\left\{ \left( \begin{array}{ll} (p -1)^2 (m-1)^2\left[ \dfrac{1+ k R^2}{R^4}\right] + \dfrac{(p -1)^2}{R^4} \\ \\ +\dfrac{ [1+\beta (p -1)]^4}{R^4} +(p-1)^2 (m-1)^2 k^2 \end{array} \right) \right. M^{4-2\beta } \nonumber \\&+ \left. \left( \begin{array}{ll} \left[ 2h^2 + \dfrac{1}{(\tau -t_0+T)^2} \right] M^{2-2\beta } + \sup _{Q_{R, T}} \left\{ \left[ \dfrac{|\Sigma _x(t,x,v)|}{v^{(3\beta -2)/2}}\right] ^{4/3}\right\} \\ \\ +\sup _{Q_{R, T}} \left\{ v^{2(1-\beta )}\left[ 2 \Sigma _v(t,x,v)-\dfrac{ \beta \Sigma (t,x,v)}{v} \right] _+^2\right\} \end{array} \right) \right\} . \end{aligned}$$

Recalling the maximality of $\eta w$ at $(x_1, t_1)$ along with $\eta \equiv 1$ when $d(x, x_0,t) \le R/2$ and $\tau \le t \le t_0$, it follows that $w^2 (x, \tau ) = (\eta ^2 w^2)(x, \tau ) \le (\eta ^2 w^2)(x_1,t_1) \le (\eta w^2)(x_1,t_1)$. Hence noting $w = |\nabla v|^2/v^{\beta }$ and absorbing $\beta$, *p* and *m* into $C(\gamma )$ gives

4.27 $$\begin{aligned} \frac{|\nabla v|}{v^{\beta /2}} (x,\tau ) \le C(p, \beta , m) \left\{ \begin{array}{ll} \sqrt{h} M^{(1-\beta )/2} + \left[ \dfrac{k^{1/4}}{\sqrt{R}} +\dfrac{1}{R} +\sqrt{k} \right] M^{1-\beta /2} \\ \\ + \dfrac{M^{(1-\beta )/2}}{\sqrt{\tau -t_0+T}} + \sup _{Q_{R, T}} \left\{ \left[ \dfrac{|\Sigma _x(t,x,v)|}{v^{(3\beta -2)/2}}\right] ^{1/3}\right\} \\ \\ + \sup _{Q_{R, T}}\left\{ v^{(1-\beta )/2}\left[ 2 \Sigma _v(t,x,v)-\dfrac{ \beta \Sigma (t,x,v)}{v} \right] _+^{1/2}\right\} \end{array} \right\} . \end{aligned}$$

The arbitrariness of $\tau$ in the interval $t_0-T<\tau \le t_0$ now gives the final conclusion. $\square$

## Ancient solutions to $$\partial _t u - \Delta _f u^p = {{\mathscr {N}}}(u)$$$$\mathbf{I}$$: proof of Theorem 2.6

Let us now present the proof of Theorem 2.6. Towards this end fix a space-time point $(x_0,t_0)$. Then with the choice of base point $(x_0,t_0)$ and $R>0$, $T=R^2$ it follows from the growth assumption $u(x,t) = o([\varrho (x) + \sqrt{|t|}]^{2/[(p-1)(2-\beta )]})$ that

5.1 $$\begin{aligned} M = \sup _{Q_{R,T}} v = [p/(p-1)] \sup _{Q_{R,T}} u^{p-1} = o(R^{1/(1-\beta /2)}). \end{aligned}$$

Next turning to the local estimate (2.5) in Theorem 2.5 (with $t=t_0$, $k=0$ and $T=R^2$) we can write

5.2 $$\begin{aligned} \frac{|\nabla v|}{v^{\beta /2}}(x_0,t_0)&\le C \left\{ \begin{array}{ll} \left[ \dfrac{k^{1/4}}{\sqrt{R}} +\dfrac{1}{R} +\sqrt{k} \right] M^{1-\beta /2} + \sup _{Q_{R, T}} \left\{ \left[ \dfrac{|\Sigma _x(t,x,v)|}{v^{(3\beta -2)/2}}\right] ^{1/3}\right\} \\ \\ + \dfrac{M^{(1-\beta )/2}}{\sqrt{T}} + \sup _{Q_{R, T}}\left\{ v^{(1-\beta )/2}\left[ 2 \Sigma _v(t,x,v)-\dfrac{ \beta \Sigma (t,x,v)}{v} \right] _+^{1/2}\right\} \end{array} \right\} \nonumber \\&\le C \left\{ \begin{array}{ll} \dfrac{M^{1-\beta /2}}{R} + \dfrac{M^{(1-\beta )/2}}{\sqrt{T}} + \sup _{Q_{R, T}} \left\{ \left[ \dfrac{|\Sigma _x(t,x,v)|}{v^{(3\beta -2)/2}}\right] ^{1/3}\right\} \\ \\ + \sup _{Q_{R, T}}\left\{ v^{(1-\beta )/2}\left[ 2 \Sigma _v(t,x,v)-\dfrac{ \beta \Sigma (t,x,v)}{v} \right] _+^{1/2}\right\} \end{array} \right\} . \end{aligned}$$

According to Remark 2.3, $\Sigma (t,x,v) = p u^{p-2} {{\mathscr {N}}}(t,x,u)$ where $u=[(p-1)v/p]^{1/(p-1)}$. Hence, in view of ${{\mathscr {N}}}={{\mathscr {N}}}(u)$, here, $\Sigma =\Sigma (v)$ and so in particular $\Sigma _x \equiv 0$. Moreover, a basic calculation based on writing $\Sigma _v=\Sigma _u \partial _v u$ where $\Sigma _u = pu^{p-2}[(p-2){{\mathscr {N}}}/u+{{\mathscr {N}}}_u]$ and $\partial _v u = u^{2-p}/p$ gives $\Sigma _v=(p-2){{\mathscr {N}}}/u+{{\mathscr {N}}}_u$. Therefore by putting the latter together,

5.3 $$\begin{aligned} 2 \Sigma _v (v) - \beta \frac{\Sigma (v)}{v}&= 2 \left[ (p-2) \frac{{{\mathscr {N}}}(u)}{u} + {{\mathscr {N}}}_u(u) \right] - \beta (p-1) \frac{{{\mathscr {N}}}(u)}{u} \nonumber \\&= [2(p-2)-\beta (p-1)] \frac{{{\mathscr {N}}}(u)}{u} + 2 {{\mathscr {N}}}_u(u) \le 0, \end{aligned}$$

where the last inequality follows from the assumptions on ${{\mathscr {N}}}$ in the theorem. Thus by virtue of (5.1) and the above calculation, we conclude from (5.2) that

5.4 $$\begin{aligned} \frac{|\nabla v|}{v^{\beta /2}}(x_0,t_0) \le C\left[ \dfrac{M^{1-\beta /2}}{R} + \dfrac{M^{(1-\beta )/2}}{\sqrt{T}} \right] \le \frac{o(R)}{R} + \frac{o(R^{(1-\beta )/(2-\beta )})}{R}. \end{aligned}$$

Now passing to the limit $R \nearrow \infty$ it follows that $|\nabla v|(x_0,t_0)=0$. The arbitrariness of $(x_0,t_0)$ implies $|\nabla v| \equiv 0$ and so *v* and subsequently *u* are spatially constant. Hence we have $u=u(t)$. From equation (2.6) satisfied by *u* it then follows that $du/dt = {{\mathscr {N}}}(u)$. Assuming now that ${{\mathscr {N}}}(u) \ge a >0$ for all $u>0$ it follows by integrating the ODE that $u(t) \le u(0) + at$ for all $t<0$. This however clashes with $u(t)>0$ as $t \searrow -\infty$ and so the conclusion is reached. $\square$

## Another Hamilton-Souplet-Zhang estimate for (1.1): $1< p < 1+1/\sqrt{m-1}$

In this section we present the second set of gradient estimates for the positive solutions to equation (1.1). The estimates as will be seen below are valid in the exponent range $1<p<1+1/\sqrt{m-1}$. We use a different set of ideas and techniques to prove these estimates, a result of which is that we can improve the key evolution inequalities used in the proof, and subsequently arrive at a larger exponent range for the validity of the estimates. (Compare with $1<p<1/(\sqrt{2m} + 1)$ in Theorem 2.1.)

### Theorem 6.1

Let $(\mathscr M, g, d\mu )$ be a complete smooth metric measure space with $d\mu =e^{-f} dv_g$. Suppose that the metric and potential are time dependent, of class $\mathscr {C}^2$ and that for suitable constants $k, h \ge 0$ and $m\ge n$ satisfy the bounds ${\mathscr {R}ic}_f^m (g) \ge -(m-1)k g$ and $\partial _t g \ge -2 h g$ in the space-time cylinder $Q_{R,T}$ with $R, T >0$. Let *u* be a positive solution to (1.1) with $1< p < 1+1/\sqrt{m-1}$ and $v =pu^{p-1}/(p-1)$ and $M=\sup _{Q_{R,T}} v$. Then there exists $C=C(p,m)>0$ such that for every (*x*, *t*) in $Q_{R/2,T}$ with $t>t_0-T$ we have

6.1 $$\begin{aligned} v^{\frac{1}{2(p-1)}}|\nabla v| (x,t) \le C \left\{ \begin{array}{ll} \sqrt{h} M^\frac{p}{2(p-1)} + \left[ \dfrac{k^{1/4}}{\sqrt{R}} + \dfrac{1}{R} + \sqrt{k} \right] M^{1+\frac{1}{2(p-1)}} \\ \\ + \sup _{Q_{R, T}}\left\{ v^{p/[2(p-1)]} \left[ 2 \Sigma _v(t,x,v)+\dfrac{\Sigma (t,x,v)}{(p-1)v} \right] _+^{1/2}\right\} \\ \\ + \dfrac{M^\frac{p}{2(p-1)}}{\sqrt{t-t_0+T}} +\sup _{Q_{R, T}} \left\{ \left[ v^{(2p+1)/[2(p-1)]} |\Sigma _x(t,x,v)| \right] ^{1/3} \right\} \end{array} \right\} . \end{aligned}$$

Subject to the bounds in Theorem 6.1 being global in space by passing to the limit $R \rightarrow \infty$ we have the following global (in space) estimate.

### Theorem 6.2

Let $(\mathscr M, g, d\mu )$ be a complete smooth metric measure space with $d\mu =e^{-f} dv_g$. Suppose that the metric and potential are time dependent, of class $\mathscr {C}^2$ and that for suitable constants $k, h \ge 0$ and $m\ge n$ satisfy the bounds ${\mathscr {R}ic}_f^m (g) \ge -(m-1)k g$ and $\partial _t g \ge -2 h g$ on $\mathscr M \times [t_0-T, t_0]$. Let *u* be a positive solution to equation (1.1) with $1< p < 1+1/\sqrt{m-1}$ and $v =pu^{p-1}/(p-1)$ and $M= \sup v$. Then there exists $C=C(p,m)>0$ such that for every $x \in \mathscr M$ and $t_0-T<t \le t_0$ we have

6.2 $$\begin{aligned} v^{\frac{1}{2(p-1)}}|\nabla v| (x,t) \le C \left\{ \begin{array}{ll} \sqrt{k} M^{1+\frac{1}{2(p-1)}} + \left[ \sqrt{h} + \dfrac{1}{\sqrt{t-t_0+T}} \right] M^\frac{p}{2(p-1)} \\ \\ +\sup _{\mathscr M \times [t_0-T, t_0]} \left\{ \left[ v^{(2p+1)/[2(p-1)]} |\Sigma _x(t,x,v)| \right] ^{1/3}\right\} \\ \\ + \sup _{\mathscr M \times [t_0-T, t_0]}\left\{ v^{p/[2(p-1)]} \left[ 2 \Sigma _v(t,x,v)+\dfrac{\Sigma (t,x,v)}{(p-1)v} \right] _+^{1/2}\right\} \end{array} \right\} . \end{aligned}$$

The special case of time independent metrics and potentials (the static case $\partial _t g \equiv 0$ and $\partial _t f \equiv 0$) is of enough significance to be formulated as a separate corollary. Here we describe the local version. The global version follows by passing to the limit $R\rightarrow \infty$.

### Theorem 6.3

Let $(\mathscr M, g, d\mu )$ be a complete smooth metric measure space with $d\mu =e^{-f} dv_g$ and assume ${\mathscr {R}ic}_f^m (g) \ge -(m-1)k g$ in ${{\mathscr {B}}}_R$ for some $k \ge 0$, $m \ge n$ and $R>0$. Let *u* be a positive solution to (1.1) with $1< p < 1+1/\sqrt{m-1}$ and $v =pu^{p-1}/(p-1)$ and $M= \sup _{Q_{R,T}} v$. Then there exists $C=C(p,m)>0$ such that for every (*x*, *t*) in $Q_{R/2,T}$ with $t>t_0-T$ we have

6.3 $$\begin{aligned} v^{\frac{1}{2(p-1)}}|\nabla v| (x,t) \le C \left\{ \begin{array}{ll} \left[ \dfrac{k^{1/4}}{\sqrt{R}} + \dfrac{1}{R} + \sqrt{k} \right] M^{1+\frac{1}{2(p-1)}} \\ \\ + \sup _{Q_{R, T}}\left\{ v^{p/[2(p-1)]} \left[ 2 \Sigma _v(t,x,v)+\dfrac{\Sigma (t,x,v)}{(p-1)v} \right] _+^{1/2}\right\} \\ \\ + \dfrac{M^\frac{p}{2(p-1)}}{\sqrt{t-t_0+T}} +\sup _{Q_{R, T}} \left\{ \left[ v^{(2p+1)/[2(p-1)]} |\Sigma _x(t,x,v)| \right] ^{1/3} \right\} \end{array} \right\} . \end{aligned}$$

We end this section with an application of the estimates above to parabolic Liouville-type theorems. Compare with Theorem 2.6.

### Theorem 6.4

Let $(\mathscr M, g, d\mu )$ be a complete smooth metric measure space with $d\mu =e^{-f}dv_g$ and ${\mathscr {R}ic}^m_f(g) \ge 0$. Assume $(3-2p) {{\mathscr {N}}}(u) - 2u {{\mathscr {N}}}_u(u) \ge 0$ for all $u>0$ where $1< p < 1+1/\sqrt{m-1}$. Then any positive ancient solution to the nonlinear porous medium equation

6.4 $$\begin{aligned} \partial _t u - \Delta _f u^p = {{\mathscr {N}}}(u(x,t)), \end{aligned}$$

satisfying the growth at infinity

6.5 $$\begin{aligned} u(x,t) = o \left( [\varrho (x) + \sqrt{|t|}]^{2/(2p-1)} \right) , \end{aligned}$$

must be spatially constant. In particular, if additionally, ${{\mathscr {N}}}(u) \ge a$ for some $a>0$ and all $u>0$ then (6.4) admits no such ancient solutions.

## Evolution inequalities $\mathbf{II}$: ${\mathscr {L}}_v^p=\partial _t - (p-1)v \Delta _f$ and $w = |\nabla v|^2/v^{\beta }$

In this section, similar to what was done earlier in Section 3, we derive evolution identities and inequalities for $w = |\nabla v|^2/v^{\beta }$ where the pressure *v* relates to the positive solution *u* via $v =pu^{p-1}/(p-1)$. The evolution operator here is ${\mathscr {L}}_v^p = \partial _t -(p-1) v \Delta _f$ and $\beta \in {{\mathbb {R}}}$ is to be specified later. Note that initially we only assume $p>1$ but later in the course of the proof of the estimates and bounds in the next section we further restrict the range of *p*.

### Lemma 7.1

Let $(\mathscr M, g, d\mu )$ be a complete smooth metric measure space with $d\mu =e^{-f} dv_g$. Suppose that the metric and potential are time dependent and of class $\mathscr {C}^2$. Let *u* be a positive solution to (1.1) and set $w = |\nabla v|^2/v^{\beta }$ where $v =pu^{p-1}/(p-1)$ and $\beta \in {{\mathbb {R}}}$ is a constant. Then for any constant $\varepsilon \in {{\mathbb {R}}}$, *w* satisfies the evolution inequality

7.1 $$\begin{aligned} {\mathscr {L}}_v^p [w]- \varepsilon \langle \nabla v, \nabla w \rangle =&~ [\partial _t-(p -1) v \Delta _f] w - \varepsilon \langle \nabla v, \nabla w\rangle \nonumber \\ \le&~\frac{\Gamma (\beta , \varepsilon )}{2 (p -1)} v^{\beta -1} w^2 - 2 \left[ \frac{1}{2} \partial _t g + (p-1) v {\mathscr {R}ic}_f^m(g) \right] \frac{(\nabla v, \nabla v)}{v^\beta } \nonumber \\&+2 \frac{\langle \nabla v, \Sigma _x(t,x,v) \rangle }{v^{\beta }} +\left[ 2 \Sigma _v(t,x,v) -\beta \frac{ \Sigma (t,x,v)}{v}\right] w. \end{aligned}$$

Here

7.2 $$\begin{aligned} \Gamma (\beta , \varepsilon ) =&~ [\varepsilon -2[1+(p -1) \beta ] -(p -1)]^2+(m-1)(p -1)^2 \nonumber \\&-2(p -1) \beta [1+(p -1)(\beta +1) -\varepsilon ]. \end{aligned}$$

### Proof

Starting from (3.5) in Lemma 3.2 and recalling ${\mathscr {L}}_v^p[w] =[\partial _t-(p -1) v \Delta _f]w$, $\Delta _f v = \Delta v - \langle \nabla f, \nabla v \rangle$ we can write

7.3 $$\begin{aligned} {\mathscr {L}}_v^p [w] =&-2\left[ \frac{1}{2}\partial _t g+(p -1)v{\mathscr {R}ic}_f^m(g)\right] \frac{( \nabla v, \nabla v)}{v^\beta } -2(p -1) \frac{|\nabla \nabla v|^2}{v^{\beta -1}}\nonumber \\&+2(p -1) \frac{|\nabla v|^2}{v^{\beta }} [\Delta v - \langle \nabla f, \nabla v \rangle ] -2\frac{p-1}{v^{\beta -1}}\frac{\langle \nabla f, \nabla v \rangle ^2}{m-n}\nonumber \\&+4[1+(p -1) \beta ] \frac{\nabla \nabla v (\nabla v, \nabla v)}{v^\beta } -\beta [1+(p -1)(\beta +1)]\frac{|\nabla v|^4}{v^{\beta +1}}\nonumber \\&+2 \frac{\langle \nabla v, \Sigma _x(t,x,v) \rangle }{v^{\beta }} +\left[ 2 \Sigma _v(t,x,v) -\beta \frac{ \Sigma (t,x,v)}{v}\right] \frac{|\nabla v|^2}{v^{\beta }}. \end{aligned}$$

Now as $\langle \nabla v, \nabla w \rangle = 2 \nabla \nabla v (\nabla v, \nabla v)/v^\beta -\beta |\nabla v|^4/v^{\beta +1}$ for any constant $\varepsilon$ we can write

7.4 $$\begin{aligned} {\mathscr {L}}_v^p [w] - \varepsilon \langle \nabla v, \nabla w \rangle =&-2(p -1) \frac{|\nabla \nabla v|^2}{v^{\beta -1}} +2(p -1) \frac{|\nabla v|^2}{v^{\beta }} \Delta v \\&- \{ 2\varepsilon -4[1+(p -1) \beta ] \} \frac{\nabla \nabla v (\nabla v, \nabla v)}{v^\beta }\nonumber \\&-2\left[ \frac{1}{2}\partial _t g+(p -1)v{\mathscr {R}ic}_f^m(g)\right] \frac{( \nabla v, \nabla v)}{v^\beta }\nonumber \\&-\frac{2(p-1)}{v^{\beta -1}} \left[ \frac{|\nabla v|^2}{v} \langle \nabla f, \nabla v \rangle +\frac{\langle \nabla f, \nabla v \rangle ^2}{(m-n)}\right] \nonumber \\&-\beta [1+(p -1)(\beta +1) -\varepsilon ]\frac{|\nabla v|^4}{v^{\beta +1}}\nonumber \\&+2 \frac{\langle \nabla v, \Sigma _x(t,x,v) \rangle }{v^{\beta }} +\left[ 2 \Sigma _v(t,x,v) -\beta \frac{ \Sigma (t,x,v)}{v}\right] \frac{|\nabla v|^2}{v^{\beta }}.\nonumber \end{aligned}$$

Next, focusing on the terms involving the second derivatives of *v* on the right-hand side, and setting $A=\nabla \nabla v$ with $\textrm{tr} A=\Delta v$ and $e = \nabla v/ |\nabla v|$, we can rewrite (7.4) as

7.5 $$\begin{aligned} \text{( }7.4\text{) } =&- 2(p -1) \frac{|A|^2}{v^{\beta -1}} +2(p -1) \frac{\textrm{tr} A }{|A|} w|A| - \{ 2\varepsilon -4[1+(p -1) \beta ] \} \frac{A(e,e)}{|A|} w |A|\nonumber \\&-2\left[ \frac{1}{2}\partial _t g+(p -1) v {\mathscr {R}ic}_f^m(g)\right] \frac{( \nabla v, \nabla v)}{v^\beta } -\beta [1+(p -1)(\beta +1) -\varepsilon ]v^{\beta -1} w^2 \nonumber \\&-\frac{2(p-1)}{v^{\beta -1}} \left[ \frac{|\nabla v|^2}{v} \langle \nabla f, \nabla v \rangle +\frac{\langle \nabla f, \nabla v \rangle ^2}{(m-n)}\right] +2 \frac{\langle \nabla v, \Sigma _x(t,x,v) \rangle }{v^{\beta }} \nonumber \\&+\left[ 2 \Sigma _v(t,x,v) -\beta \frac{ \Sigma (t,x,v)}{v}\right] w. \end{aligned}$$

Rearranging the expression on the first line then results in

7.6 $$\begin{aligned} \text{( }7.4\text{) } =&- 2(p -1) \frac{|A|^2}{v^{\beta -1}} -\left[ \{ 2\varepsilon -4[1+(p -1) \beta ] \} \frac{A(e,e)}{|A|} -2(p -1) \frac{\textrm{tr} A }{|A|} \right] w |A| \nonumber \\&-2\left[ \frac{1}{2}\partial _t g+(p -1) v {\mathscr {R}ic}_f^m(g)\right] \frac{( \nabla v, \nabla v)}{v^\beta } -\beta [1+(p -1)(\beta +1) -\varepsilon ] v^{\beta -1} w^2 \nonumber \\&-\frac{2(p-1)}{v^{\beta -1}} \left[ \frac{|\nabla v|^2}{v} \langle \nabla f, \nabla v \rangle +\frac{\langle \nabla f, \nabla v \rangle ^2}{(m-n)}\right] +2 \frac{\langle \nabla v, \Sigma _x(t,x,v) \rangle }{v^{\beta }}\nonumber \\&+\left[ 2 \Sigma _v(t,x,v) -\beta \frac{ \Sigma (t,x,v)}{v}\right] w. \end{aligned}$$

Now basic algebraic manipulations involving completing squares leads to

7.7 $$\begin{aligned} {\mathscr {L}}_v^p&[w] - \varepsilon \langle \nabla v, \nabla w \rangle =[\partial _t-(p - 1) v \Delta _f]w - \varepsilon \langle \nabla v, \nabla w \rangle \nonumber \\ =&- 2(p -1) \left[ \frac{|A|}{v^{\frac{\beta -1}{2}}} + \frac{1}{2(p -1)} \left( \{\varepsilon -2[1+(p -1) \beta ] \} \frac{A(e,e)}{|A|} -(p -1) \frac{\textrm{tr} A }{|A|} \right) v^{\frac{\beta -1}{2}} w \right] ^2 \nonumber \\&+\frac{1}{2(p -1)} \left[ \{\varepsilon -2[1+(p -1) \beta ] \} \frac{A(e,e)}{|A|} -(p -1) \frac{\textrm{tr} A }{|A|}\right] ^2 v^{\beta -1} w^2\nonumber \\&-2\left[ \frac{1}{2}\partial _t g +(p -1)v{\mathscr {R}ic}_f^m(g)\right] \frac{( \nabla v, \nabla v)}{v^\beta } -\frac{2(p-1)}{v^{\beta -1}} \left[ \frac{\langle \nabla f, \nabla v \rangle }{\sqrt{m-n}} + \frac{|\nabla v|^2 }{2v} \sqrt{m-n}\right] ^2 \nonumber \\&+ \frac{(p -1)(m-n)}{2} v^{\beta -1} w^2 -\beta [1+(p -1)(\beta +1) -\varepsilon ] v^{\beta -1} w^2\nonumber \\&+2 \frac{\langle \nabla v, \Sigma _x(t,x,v) \rangle }{v^{\beta }} +\left[ 2 \Sigma _v(t,x,v) -\beta \frac{ \Sigma (t,x,v)}{v}\right] w. \end{aligned}$$

Subsequently, as we are aiming for an upper bound, ignoring non-positive terms lead to the inequality

7.8 $$\begin{aligned} {\mathscr {L}}_v^p [w] - \varepsilon \langle \nabla v, \nabla w \rangle&\le \frac{1}{2 (p -1)} \left[ \{ \varepsilon -2[1+(p -1) \beta ] \} \frac{A(e,e)}{|A|} -(p -1) \frac{\textrm{tr} A}{|A|}\right] ^2 v^{\beta -1} w^2\nonumber \\&-2\left[ \frac{1}{2}\partial _t g+(p -1) v {\mathscr {R}ic}_f^m(g)\right] \frac{( \nabla v, \nabla v)}{v^\beta } + \frac{(p -1)(m-n)}{2} v^{\beta -1} w^2 \nonumber \\&-\beta [1+(p -1)(\beta +1) -\varepsilon ] v^{\beta -1} w^2 +2 \frac{\langle \nabla v, \Sigma _x(t,x,v) \rangle }{v^{\beta }}\nonumber \\&+\left[ 2 \Sigma _v(t,x,v) -\beta \frac{ \Sigma (t,x,v)}{v}\right] w. \end{aligned}$$

Now to proceed further we make use of the following standard matrix variational result.

### Lemma 7.2

For any $a, b \in {{\mathbb {R}}}$ we have

7.9 $$\begin{aligned} \max _{\begin{array}{c} A \in \mathbf{Sym}_n \\ A \ne 0 \\ {|e|=1} \end{array}} \left[ \frac{aA + b(\textrm{tr} A)I_n}{|A|} (e,e) \right] ^2 = (a+b)^2 +(n-1) b^2. \end{aligned}$$

Here $\mathbf{Sym}_n$ is the space of real symmetric matrices and $I_n$ the identity matrix.

Applying Lemma 7.2 to maximise the expression on the first line of inequality (7.8) (with the choices of $a= \varepsilon -2[1+(p -1) \beta ]$ and $b= -(p -1)$) and rearranging terms results in

7.10 $$\begin{aligned} {\mathscr {L}}_v^p [w] - \varepsilon \langle \nabla v, \nabla w \rangle \le&~ \frac{1}{2 (p -1)} \bigg \{ [\varepsilon -2[1+(p -1) \beta ] -(p -1)]^2 + (n-1)(p -1)^2 \nonumber \\&-2(p -1) \beta [1+(p -1)(\beta +1) -\varepsilon ] + (p -1)^2(m-n)\bigg \} v^{\beta -1} w^2 \nonumber \\&-2\left[ \frac{1}{2}\partial _t g+(p -1)v{\mathscr {R}ic}_f^m(g)\right] \frac{( \nabla v, \nabla v)}{v^\beta } +2 \frac{\langle \nabla v, \Sigma _x(t,x,v) \rangle }{v^{\beta }}\nonumber \\&+\left[ 2 \Sigma _v(t,x,v) -\beta \frac{ \Sigma (t,x,v)}{v}\right] w. \end{aligned}$$

Simplifying further leads to the desired conclusion. $\square$

### Lemma 7.3

Under the assumptions of Lemma 7.1, if the metric $g$ and potential $f$ satisfy the super flow inequality

7.11 $$\begin{aligned} \frac{1}{2} \partial _t g + (p-1) v {\mathscr {R}ic}_f^m (g) \ge - {{\textsf{k}}} g, \end{aligned}$$

then *w* satisfies the evolution inequality

7.12 $$\begin{aligned} {\mathscr {L}}_v^p [w]- p \langle \nabla v, \nabla w \rangle \le&~ \frac{(m-1)(p -1)^2-1}{2(p -1)} \frac{w^2}{v^{p/(p-1)}} + 2{{\textsf{k}}} w \\&+2 v^{1/(p-1)}\langle \nabla v, \Sigma _x(t,x,v) \rangle +\left[ 2 \Sigma _v(t,x,v) + \frac{ \Sigma (t,x,v)}{(p-1)v}\right] w. \nonumber \end{aligned}$$

Note that here may be a constant or more generally a function of space and time. Moreover, for the coefficient of the term $v^{\beta -1} w^2= w^2/v^{p/(p-1)}$ (see also (7.14) below) to be negative (i.e., $<0$) it suffices to have $1<p<1+1/\sqrt{m-1}$. The significance of this negativity will become apparent later (*see*, e.g., (8.4) in the next section).

### Proof

Optimising the quadratic function $\Gamma (\beta , \varepsilon )$ defined by (7.2) in the inequality (7.1) leads to the optimiser $(\beta ^\star , \varepsilon ^\star )$ with

7.13 $$\begin{aligned} \beta ^\star = -\frac{1}{p-1}, \qquad \text {and} \qquad \varepsilon ^\star = p. \end{aligned}$$

Substituting these into (7.2) then leads to the optimal value

7.14 $$\begin{aligned} \Gamma (\beta ^\star , \varepsilon ^\star ) = \Gamma (-1/(p-1), p) = (m-1)(p-1)^2-1. \end{aligned}$$

Using the optimal values (7.13) and (7.14) in the inequality (7.1) with $w=|\nabla v|^2 /v^\beta = v^{1/(p-1)}|\nabla v|^2$ then results in

8.1 $$\begin{aligned} {\mathscr {L}}_v^p [w]- p \langle \nabla v, \nabla w \rangle \le&~ \frac{(m-1)(p -1)^2-1}{2(p -1)} \frac{w^2}{v^{p/(p-1)}} \nonumber \\&- 2 v^{1/(p-1)} \left[ \frac{1}{2} \partial _t g+(p -1) v {\mathscr {R}ic}_f^m(g) \right] ( \nabla v, \nabla v) \nonumber \\&+2 v^{1/(p-1)}\langle \nabla v, \Sigma _x(t,x,v) \rangle +\left[ 2 \Sigma _v(t,x,v) + \frac{ \Sigma (t,x,v)}{(p-1)v}\right] w.\nonumber \end{aligned}$$

Invoking the super flow inequality (7.11) gives at once the desired conclusion. $\square$

## Cylindrical localisation and the proof of Theorem 6.1

Starting again with the localised function $\eta w$, where $\eta$ is as in (4.2), it follows upon using the identity in Lemma 4.2 that

8.2 $$\begin{aligned} {\mathscr {L}}_v^p[\eta w] = w{\mathscr {L}}_v^p[\eta ] - 2(p -1)v [\langle \nabla \eta ,\nabla (\eta w) \rangle - |\nabla \eta |^2 w]/\eta +\eta {\mathscr {L}}_v^p [w]. \end{aligned}$$

Using (8.1) along with the observation that $\eta \langle \nabla v, \nabla w \rangle = \langle \nabla v, \nabla (\eta w) \rangle - w \langle \nabla v, \nabla \eta \rangle$ then gives

8.3 $$\begin{aligned} {\mathscr {L}}_v^p [\eta w] \le&~ w {\mathscr {L}}_v^p [\eta ] -2(p -1) v \left\langle \frac{\nabla \eta }{\eta } , \nabla (\eta w) \right\rangle + 2(p -1)v \frac{|\nabla \eta |^2}{\eta } w \nonumber \\&+\varepsilon ^\star \langle \nabla v , \nabla (\eta w) \rangle -\varepsilon ^\star w\langle \nabla v , \nabla \eta \rangle +\frac{\Gamma (\beta ^\star , \varepsilon ^\star )}{2 (p -1)} v^{\beta ^\star -1} \eta w^2 \nonumber \\&+ 2[(p - 1)(m-1)k v+h] \eta w + 2\eta \frac{\langle \nabla v, \Sigma _x(t,x,v) \rangle }{v^{\beta ^\star }} \nonumber \\&+ \left[ 2 \Sigma _v(t,x,v) -\frac{ \beta ^\star \Sigma (t,x,v)}{v} \right] \eta w. \end{aligned}$$

Assume that the localised function $\eta w$ is maximised at the point $(x_1, t_1)$ in the compact space-time set $\{d(x,x_0, t) \le R, t_0-T \le t \le \tau \}$. Additionally, we can assume that $(\eta w)(x_1, t_1) >0$ as otherwise the conclusion of the theorem is true with $w(x, \tau ) \le 0$ for all $d(x, x_0, \tau ) \le R/2$. Hence $t_1>t_0-T$ and at the point $(x_1,t_1)$ we have $\Delta _f(\eta w) \le 0$, $\partial _t (\eta w) \ge 0$ and $\nabla (\eta w) =0$. Thus at the point $(x_1,t_1)$ we also have ${\mathscr {L}}_v^p [\eta w] \ge 0$. From (8.2) upon rearranging of terms it therefore follows that

8.4 $$\begin{aligned} - \frac{\Gamma (\beta ^\star , \varepsilon ^\star )}{2 (p -1)} v^{\beta ^\star -1} \eta w^2 \le&~ w {\mathscr {L}}_v^p [\eta ] +2[(p - 1)(m-1) k v + h] \eta w\nonumber \\&- \varepsilon ^\star w\langle \nabla v , \nabla \eta \rangle + 2(p -1)v \frac{|\nabla \eta |^2}{\eta } w\nonumber \\&+\left[ 2 \Sigma _v(t,x,v)-\frac{ \beta ^\star \Sigma (t,x,v)}{v} \right] \eta w + 2\eta \frac{\langle \nabla v, \Sigma _x(t,x,v) \rangle }{v^{\beta ^\star }}. \end{aligned}$$

Denoting $-\Gamma (\beta ^\star , \varepsilon ^\star )/[2(p -1)]= 2/\gamma >0$, that is, $\gamma =4(p-1)/[1-(m-1) (p-1)^2]$ (see the comments following Lemma 7.3) and multiplying through by $\gamma v^{1-\beta ^\star }$ gives

8.5 $$\begin{aligned} 2\eta w^2 \le&~ \gamma v^{1-\beta ^\star } w {\mathscr {L}}_v^p [\eta ] +2 \gamma [(p - 1)(m-1) k_1 v + k_2]v^{1-\beta ^\star } \eta w\nonumber \\&- \varepsilon ^\star \gamma v^{1-\beta ^\star } w\langle \nabla v , \nabla \eta \rangle + 2 \gamma (p -1)v^{2-\beta ^\star } \frac{|\nabla \eta |^2}{\eta } w\nonumber \\&+\gamma v^{1-\beta ^\star } \eta w \left[ 2 \Sigma _v(t,x,v)-\frac{ \beta ^\star \Sigma (t,x,v)}{v} \right] + 2 \gamma \eta v^{1-\beta ^\star } \frac{\langle \nabla v, \Sigma _x(t,x,v) \rangle }{v^{\beta ^\star }}. \end{aligned}$$

Now we proceed onto bounding the expression on the right-hand side of (8.4). As this is similar to the proof of Theorem 2.1 we shall remain brief, focusing mainly on the differences.

Towards this end, starting from the first term on the right and arguing as in Theorem 2.1 we have

8.6 $$\begin{aligned} \gamma v^{1-\beta ^\star } w {\mathscr {L}}_v^p [\eta ] \le&~ \frac{1}{7} \eta w^2 + C \gamma ^2 \left[ \frac{1}{(\tau -t_0+T)^2} + h^2\right] (\sup _{Q_{R, T}} v)^{2-2\beta ^\star }\nonumber \\&+ C \gamma ^2(p -1)^2 (m-1)^2\left[ \frac{1+ k R^2}{R^4}\right] (\sup _{Q_{R, T}} v)^{4-2\beta ^\star }. \end{aligned}$$

Likewise for the second term we can write

8.7 $$\begin{aligned} 2 \gamma (p - 1)(m-1) k v^{2-\beta ^\star } \eta w \le \frac{1}{7} \eta w^2 + C \gamma ^2 (p-1)^2 (m-1)^2 k^2(\sup _{Q_{R, T}} v)^{4-2\beta ^\star }, \\ 2 \gamma h v^{1-\beta ^\star } \eta w \le 2 \gamma h v^{1-\beta ^\star } \eta ^{1/2} w \le \frac{1}{7} \eta w^2 + C \gamma ^2 h^2 (\sup _{Q_{R, T}} v)^{2-2\beta ^\star }. \end{aligned}$$

We can bound the next term by writing

8.8 $$\begin{aligned} -\varepsilon ^\star \gamma v^{1-\beta ^\star }w \langle \nabla v , \nabla \eta \rangle&\le \varepsilon ^\star \gamma v^{1-\beta ^\star }w |\nabla v| |\nabla \eta | \nonumber \\&\le \varepsilon ^\star \gamma v^{1-\beta ^\star /2} \eta ^{3/4} w^{3/2} \frac{|\nabla \eta |}{\eta ^{3/4}}\nonumber \\&\le \frac{1}{7} (\eta ^{3/4} w^{3/2})^{4/3} + C \varepsilon ^{\star 4} \gamma ^4 \left[ \frac{|\nabla \eta |}{\eta ^{3/4}}\right] ^4 v^{4-2\beta ^\star } \nonumber \\&\le \frac{1}{7} \eta w^2 + \frac{C \varepsilon ^{\star 4} \gamma ^4}{R^4} (\sup _{Q_{R, T}} v)^{4-2\beta ^\star }. \end{aligned}$$

In much the same way

8.9 $$\begin{aligned} 2 \gamma (p -1) v^{2-\beta ^\star } \frac{|\nabla \eta |^2}{\eta } w \le \frac{1}{7} \eta w^2 + \frac{C \gamma ^2 (p -1)^2}{R^4}(\sup _{Q_{R, T}} v)^{4-2\beta ^\star }. \end{aligned}$$

Finally for the $\Sigma (t,x,v)$ terms, upon recalling $0\le \eta \le 1$ and utilising Young’s inequality we have

8.10 $$\begin{aligned} \gamma v^{1-\beta ^\star }\eta w \bigg [2 \Sigma _v(t,x,v)&-\frac{ \beta ^\star \Sigma (t,x,v)}{v} \bigg ]\\&\le \frac{1}{7} \eta w^2 + C \gamma ^2 \sup _{Q_{R, T}} \left\{ v^{2(1-\beta ^\star )}\left[ 2 \Sigma _v(t,x,v)-\frac{ \beta ^\star \Sigma (t,x,v)}{v} \right] _+^2\right\} .\nonumber \end{aligned}$$

Furthermore, by using Cauchy-Schwarz and Young inequalities we can write

8.11 $$\begin{aligned} 2 \gamma v^{1-\beta ^\star } \eta \frac{\langle \nabla v, \Sigma _x(t,x,v) \rangle }{v^{\beta ^\star }} \le&~ 2 \gamma v^{1-\beta ^\star } \eta |\nabla v| \frac{|\Sigma _x(t,x,v)|}{v^{\beta ^\star }}\\ \le&~ \frac{1}{7} \eta w^2+C \gamma ^{4/3} \sup _{Q_{R, T}} \left\{ \left[ \frac{|\Sigma _x(t,x,v)|}{v^{(3\beta ^\star -2)/2}}\right] ^{4/3}\right\} .\nonumber \end{aligned}$$

Completing the estimate of the individual terms on the right-hand side of (8.4) we now proceed by substituting these back into the inequality. Recalling the explicit optimal values (7.13) and using the local bound $M=\sup _{Q_{R,T}} v$ it then follows from (8.4) and the individual bounds above (note that the coefficient 2 of $\eta w^2$ on the left-hand side exceeds the sum 1 of the coefficients of the seven term involving $\eta w^2$ on the right-hand side where each coefficient appears as 1/7) that

8.12 $$\begin{aligned} \eta w^2 \le C(\gamma )&\left\{ \left( \begin{array}{ll} (p -1)^2 (m-1)^2\left[ \dfrac{1+ k R^2}{R^4}\right] +\dfrac{p^4}{R^4} \\ \\ +\dfrac{(p -1)^2}{R^4} + (p-1)^2 (m-1)^2 k^2 \end{array} \right) \right. M^{4+2/(p-1)} \nonumber \\&\qquad + \left. \left( \begin{array}{ll} \left[ 2h^2 + \dfrac{1}{(\tau -t_0+T)^2} \right] M^{2+2/(p-1)} \\ \\ \sup _{Q_{R, T}} \left\{ v^{2p/(p-1)} \left[ 2 \Sigma _v(t,x,v)+\dfrac{\Sigma (t,x,v)}{(p-1)v} \right] _+^2\right\} \\ \\ + \sup _{Q_{R, T}} \left\{ [v^{(2p+1)/[2(p-1)]}|\Sigma _x(t,x,v)|]^{4/3}\right\} \end{array} \right) \right\} . \end{aligned}$$

Recalling the maximality of $\eta w$ at $(x_1, t_1)$ along with $\eta \equiv 1$ when $d(x, x_0,t) \le R/2$ and $\tau \le t \le t_0$, it follows that $w^2 (x, \tau ) = (\eta ^2 w^2)(x, \tau ) \le (\eta ^2 w^2)(x_1,t_1) \le (\eta w^2)(x_1,t_1)$. Hence noting $w = v^{1/(p-1)}|\nabla v|^2$ and absorbing *p* and *m* into $C(\gamma )$ gives

8.13 $$\begin{aligned} {[}v^{1/[2(p-1)]}|\nabla v|] (x,\tau ) \le C \left\{ \begin{array}{ll} \sqrt{h} M^\frac{p}{2(p-1)} + \left[ \dfrac{k^{1/4}}{\sqrt{R}} +\dfrac{1}{R} +\sqrt{k} \right] M^{1+\frac{1}{2(p-1)}} \\ \\ + \sup _{Q_{R, T}}\left\{ v^\frac{p}{2(p-1)} \left[ 2 \Sigma _v(t,x,v)+\dfrac{\Sigma (t,x,v)}{(p-1)v} \right] _+^{1/2}\right\} \\ \\ + \dfrac{M^\frac{p}{2(p-1)}}{\sqrt{\tau -t_0+T}} + \sup _{Q_{R, T}} \left\{ [v^\frac{2p+1}{2(p-1)} |\Sigma _x(t,x,v)|]^{1/3}\right\} \end{array} \right\} . \end{aligned}$$

The arbitrariness of $t_0-T<\tau \le t_0$ now gives the desired conclusion. $\square$

## Ancient solutions to $$\partial _t u - \Delta _f u^p = {{\mathscr {N}}}(u)$$$$\mathbf{II}$$: proof of Theorem 6.4

Let us now present the proof of Theorem 6.4. We first fix a space-time point $(x_0,t_0)$. Then with the choices $t=t_0$, $R>0$ and $T=R^2$ it follows from the growth assumption $u(x,t) = o([\varrho (x) + \sqrt{|t|}]^{2/(2p-1)})$ that for *R* sufficiently large

9.1 $$\begin{aligned} M = \sup _{Q_{R,T}} v = [p/(p-1)] \sup _{Q_{R,T}} u^{p-1} = o(R^\frac{p-1}{p-1/2}). \end{aligned}$$

Now turning to the local estimate (6.3) in Theorem 6.3 (with $k=0$) we can write

9.2 $$\begin{aligned} v^{\frac{1}{2(p-1)}}|\nabla v| (x_0,t_0)&\le C \left\{ \begin{array}{ll} \left[ \dfrac{k^{1/4}}{\sqrt{R}} +\dfrac{1}{R} +\sqrt{k} \right] M^{1+\frac{1}{2(p-1)}} \\ \\ + \sup _{Q_{R, T}}\left\{ v^\frac{p}{2(p-1)} \left[ 2 \Sigma _v(t,x,v)+\dfrac{\Sigma (t,x,v)}{(p-1)v} \right] _+^\frac{1}{2}\right\} \\ \\ + \dfrac{M^\frac{p}{2(p-1)}}{\sqrt{\tau -t_0+T}} + \sup _{Q_{R, T}} \left\{ [v^\frac{2p+1}{2(p-1)} |\Sigma _x(t,x,v)|]^\frac{1}{3}\right\} \end{array} \right\} \nonumber \\&\le C \left\{ \begin{array}{ll} \dfrac{M^{1+\frac{1}{2(p-1)}}}{R} +\sup _{Q_{R, T}} \left\{ v^\frac{2p+1}{2(p-1)} \left[ |\Sigma _x(t,x,v)| \right] ^\frac{1}{3} \right\} \\ \\ \dfrac{M^{\frac{p}{2(p-1)}} }{\sqrt{T}} + \sup _{Q_{R, T}}\left\{ v^\frac{p}{2(p-1)} \left[ 2 \Sigma _v(t,x,v)+\dfrac{\Sigma (t,x,v)}{(p-1)v} \right] _+^\frac{1}{2}\right\} \end{array} \right\} . \end{aligned}$$

Next referring to Remark 2.3 we have $\Sigma (v) = p u^{p-2} {{\mathscr {N}}}(u)$ where $u=[(p-1)v/p]^{1/(p-1)}$. Thus in particular $\Sigma _x \equiv 0$ and $\Sigma _v=\Sigma _u \partial _v u =(p-2){{\mathscr {N}}}/u+{{\mathscr {N}}}_u$. Hence

$$\begin{aligned} 2 \Sigma _v(v) + \frac{\Sigma (v)}{(p-1)v}&= 2 \left[ (p-2) \frac{{{\mathscr {N}}}(u)}{u} + {{\mathscr {N}}}_u(u) \right] + \frac{{{\mathscr {N}}}(u)}{u} \nonumber \\&= [2(p-2) +1] \frac{{{\mathscr {N}}}(u)}{u} + 2 {{\mathscr {N}}}_u(u) \le 0, \end{aligned}$$

where the last inequality follows from the assumptions on ${{\mathscr {N}}}$ in the theorem. Hence by virtue of (9.1) we deduce from (9.2) that

9.3 $$\begin{aligned} v^{\frac{1}{2(p-1)}}|\nabla v| (x_0,t_0) \le \dfrac{M^{1+\frac{1}{2(p-1)}} }{R} + \dfrac{M^{\frac{p}{2(p-1)}} }{\sqrt{T}} \le \frac{o(R)}{R} + \frac{o(R^\frac{p}{2p-1})}{R}. \end{aligned}$$

Passing to the limit $R \nearrow \infty$ and noting that $p/(2p-1) \le 1$ in the range $p>1$ it follows that $|\nabla v|(x_0,t_0)=0$. The arbitrariness of $(x_0,t_0)$ implies $|\nabla v| \equiv 0$ and so *v* and subsequently *u* are spatially constant. Hence we have $u=u(t)$ and from equation (6.4) it follows that $du/dt = {{\mathscr {N}}}(u)$. The rest is similar to the proof of Theorem 2.6. $\square$

## A Ricci-Perelman super flow inequality $\partial _t g + 2(p-1) v {\mathscr {R}ic}^m_f(g) \ge - 2{\textsf k} g$

In this final section of the paper we derive a global gradient bound on positive smooth solutions to equation (1.1). Here $\mathscr M$ is closed and the metric and potential are assumed to evolve under the super flow inequality

10.1 $$\begin{aligned} {\left\{ \begin{array}{ll} \dfrac{1}{2} \dfrac{\partial g}{\partial t} (x,t) + (p-1) v(x,t) {\mathscr {R}ic}^m_f(g)(x,t) \ge - {\textsf k} g(x,t), \\ \\ {\mathscr {R}ic}_f^m(g)(x,t) = {\mathscr {R}ic} (g)(x,t) + \nabla _{g} \nabla _{g} f (x,t) \\ \qquad \qquad \qquad \quad - \dfrac{\nabla _{g} f(x,t) \otimes \nabla _{g} f(x,t)}{m-n}. \end{array}\right. } \end{aligned}$$

Here $p>1$ but we will refine this range further later on. (See Corollaries 10.5 and 10.6.)

### Lemma 10.1

Let *u* be a positive smooth solution to (1.1) and set $v =pu^{p-1}/(p-1)$. For $s \ge 2$, $q \in {{\mathbb {R}}}$ and $\zeta =\zeta (t)$ non-negative and of class ${{\mathscr {C}}}^1[0, \infty )$ let

10.2 $$\begin{aligned} {{\textsf{H}}}^{s,q}_\zeta [v] = \zeta (t) \frac{|\nabla v|^s}{v^q} + \Gamma (v), \end{aligned}$$

where $\Gamma =\Gamma (v)$ with $v>0$ is of class $\mathscr {C}^2$. Then, for every $\varepsilon \in {{\mathbb {R}}}$, ${{\textsf{H}}} = {{\textsf{H}}}^{s, q}_\zeta [v]$ satisfies the evolution identity,

10.3 $$\begin{aligned} {{\mathscr {L}}}_v^{p} ({{\textsf{H}}}^{s, q}_\zeta [v])&- \varepsilon \langle \nabla v, \nabla {{\textsf{H}}}^{s, q}_\zeta [v] \rangle = [\partial _t -(p-1) v \Delta _ f] ({{\textsf{H}}}^{s, q}_\zeta [v]) - \varepsilon \langle \nabla v, \nabla {{\textsf{H}}}^{s, q}_\zeta [v] \rangle \nonumber \\ =&~ \zeta '(t) \frac{|\nabla v|^s}{v^q} - s \zeta (t) \frac{|\nabla v|^{s-2}}{v^q} \left[ \frac{1}{2} \partial _t g + (p-1) v {\mathscr {R}ic}_f^m (g)\right] (\nabla v, \nabla v) \nonumber \\&+ \zeta (t) \frac{|\nabla v|^{s-2}}{v^q} \left\{ s (p-1) \left[ |\nabla v|^2 \Delta _f v - v |\nabla \nabla v|^2 - v \frac{\langle \nabla f , \nabla v \rangle ^2}{(m-n)} \right] \right. \nonumber \\&\left. + \left[ s[q(p-1)+1-\varepsilon /2] \langle \nabla |\nabla v|^2, \nabla v\rangle - q[q(p-1) +p-\varepsilon ] \frac{|\nabla v|^{4}}{v}\right] \right\} \nonumber \\&- \zeta (t)\frac{s(p-1)}{2v^{q-1}} \langle \nabla |\nabla v|^{s-2} , \nabla |\nabla v|^2 \rangle + s \zeta (t) \frac{|\nabla v|^{s-2}}{v^q}\langle \nabla v, \nabla \Sigma (t,x,v) \rangle \nonumber \\&- q \zeta (t)\frac{|\nabla v|^s}{v^q} \frac{\Sigma (t,x,v)}{v} - (p-1)v\Gamma ''(v) |\nabla v|^2\nonumber \\&+ \Gamma '(v) [(1-\varepsilon )|\nabla v|^2 +\Sigma (t,x,v)]. \end{aligned}$$

### Proof

Referring to (10.2) it is a straightforward matter to see that

10.4 $$\begin{aligned} {{\mathscr {L}}}_v^{p} ({{\textsf{H}}}^{s,q}_\zeta [v]) = \zeta '(t) \frac{|\nabla v|^s}{v^q} + \zeta (t) {{\mathscr {L}}}_v^{p} \left[ \frac{|\nabla v|^s}{v^q} \right] + {{\mathscr {L}}}_v^{p} [\Gamma (v)]. \end{aligned}$$

We need to evaluate the expressions in the second and third terms on the right-hand side respectively. Focusing on the second term, first it is easily seen that,

10.5 $$\begin{aligned} {{\mathscr {L}}}_v^{p} \left[ \frac{|\nabla v|^s}{v^q} \right] =&~ \frac{1}{v^q} {{\mathscr {L}}}_v^{p} [|\nabla v|^s] + 2 (p-1) v \left\langle \nabla \left( \frac{|\nabla v|^s}{v^q}\right) , \frac{\nabla v^q}{v^q} \right\rangle - \frac{|\nabla v|^s}{v^{2q}} {{\mathscr {L}}}_v^{p} [v^q]. \end{aligned}$$

Now for the first term on the right-hand side of (10.5) a direct calculation gives

10.6 $$\begin{aligned} {{\mathscr {L}}}_v^{p} [|\nabla v|^s] =&~ [\partial _t -(p-1) v \Delta _ f] |\nabla v|^s = \partial _t (|\nabla v|^2)^{s/2} -(p-1) v \Delta _ f (|\nabla v|^2)^{s/2} \nonumber \\ =&~\frac{s}{2} |\nabla v|^{s-2} \left[ \partial _t -(p-1)v \Delta _f\right] |\nabla v|^2 - \frac{s}{2}(p-1)v \langle \nabla |\nabla v|^{s-2} , \nabla |\nabla v|^2 \rangle \nonumber \\ =&~ \frac{s}{2} |\nabla v|^{s-2} {{\mathscr {L}}}_v^{p} [|\nabla v|^2] - \frac{s}{2}(p-1)v \langle \nabla |\nabla v|^{s-2} , \nabla |\nabla v|^2 \rangle , \end{aligned}$$

whilst using Lemma 3.1 and the weighted Bochner-Weitzenböck formula (1.8) we have

10.7 $$\begin{aligned} {\mathscr {L}}^p_v [|\nabla v|^2] =&-[\partial _t g] ( \nabla v, \nabla v) +2 (p-1) |\nabla v|^2 \Delta _f v + 2\langle \nabla v, \nabla |\nabla v|^2 \rangle \nonumber \\&+2 \langle \nabla v, \nabla \Sigma (t,x,v) \rangle -2(p-1) v |\nabla \nabla v|^2\nonumber \\&-2(p-1)v {\mathscr {R}ic}_f^m (\nabla v, \nabla v) - 2 (p-1) v \langle \nabla f , \nabla v \rangle ^2/(m-n). \end{aligned}$$

Thus combining the two identities above it follows that

10.8 $$\begin{aligned} {{\mathscr {L}}}_v^{p} [|\nabla v|^s] =&~ \frac{s}{2} |\nabla v|^{s-2} {{\mathscr {L}}}_v^{p} [|\nabla v|^2] - \frac{s}{2}(p-1)v \langle \nabla |\nabla v|^{s-2} , \nabla |\nabla v|^2 \rangle \nonumber \\ =&-\frac{s}{2} |\nabla v|^{s-2} [\partial _t g] ( \nabla v, \nabla v) + s (p-1) |\nabla v|^s \Delta _f v + s |\nabla v|^{s-2}\langle \nabla v, \nabla |\nabla v|^2 \rangle \nonumber \\&+ s |\nabla v|^{s-2}\langle \nabla v, \nabla \Sigma (t,x,v) \rangle - s (p-1) v |\nabla v|^{s-2} |\nabla \nabla v|^2\nonumber \\&- s (p-1)v |\nabla v|^{s-2} {\mathscr {R}ic}_f^m (\nabla v, \nabla v) -s (p-1)v |\nabla v|^{s-2} \langle \nabla f , \nabla v \rangle ^2/(m-n)\nonumber \\&-\frac{s}{2}(p-1)v \langle \nabla |\nabla v|^{s-2} , \nabla |\nabla v|^2 \rangle . \end{aligned}$$

Next, for the second term on the right-hand side of (10.5) we can write

10.9 $$\begin{aligned} \left\langle \nabla \left( \frac{|\nabla v|^s}{v^q}\right) , \frac{\nabla v^q}{v^q} \right\rangle =&~\frac{sq}{2} \frac{|\nabla v|^{s-2}}{v^{q+1}}\langle \nabla |\nabla v|^2 , \nabla v\rangle - q^2 \frac{|\nabla v|^{s+2}}{v^{q+2}}. \end{aligned}$$

In a similar way for the last term on the right-hand side of (10.5) we have

10.10 $$\begin{aligned} {{\mathscr {L}}}_v^{p} [v^q]&=q v^{q-1} \partial _t v -(p-1) v q v^{q-1} \Delta _f v -(p-1) q(q-1) v^{q-1} |\nabla v|^2\nonumber \\&=q v^{q-1} [\partial _t - (p-1) v \Delta _f] v -(p-1) q(q-1) v^{q-1} |\nabla v|^2\nonumber \\&=q v^{q-1} {{\mathscr {L}}}_v^{p} [v] -(p-1) q(q-1) v^{q-1} |\nabla v|^2 \nonumber \\&=q v^{q-1} |\nabla v|^2+ q v^{q-1}\Sigma (t,x,v) -(p-1)q(q-1) v^{q-1} |\nabla v|^2, \end{aligned}$$

where in concluding the last line we have used (3.1). Substituting (10.8), (10.9) and (10.10) back into (10.5) and rearranging terms now leads to

10.11 $$\begin{aligned} {{\mathscr {L}}}_v^{p} \left[ \frac{|\nabla v|^s}{v^q} \right] =&- s \frac{|\nabla v|^{s-2}}{v^q} \left[ \frac{1}{2} \partial _t g + (p-1) v {\mathscr {R}ic}_f^m(g) \right] (\nabla v, \nabla v) - \frac{q}{v^{q+1}} |\nabla v|^s\Sigma (t,x,v) \nonumber \\&+ s (p-1) \frac{|\nabla v|^{s-2}}{v^q} \left[ |\nabla v|^2 \Delta _f v - v |\nabla \nabla v|^2 - v \frac{\langle \nabla f , \nabla v \rangle ^2}{(m-n)} \right] \nonumber \\&+ s \frac{|\nabla v|^{s-2}}{v^{q-1}} \left[ \frac{1}{v} [q(p-1)+1] \langle \nabla |\nabla v|^2 , \nabla v\rangle - \frac{q}{s}[q(p-1) +p] \frac{|\nabla v|^{4}}{v^2}\right] \nonumber \\&-\frac{s}{2v^{q-1}}(p-1) \langle \nabla |\nabla v|^{s-2} , \nabla |\nabla v|^2 \rangle + s \frac{|\nabla v|^{s-2}}{v^q}\langle \nabla v, \nabla \Sigma (t,x,v) \rangle . \end{aligned}$$

This completes the calculation of the second term on the right-hand side of (10.4). Since for the last term of the same equation we can write

10.12 $$\begin{aligned} {{\mathscr {L}}}_v^{p} [\Gamma (v)]&= \Gamma '(v) [|\nabla v|^2 + \Sigma (t,x,v)] - (p-1)v\Gamma ''(v) |\nabla v|^2, \end{aligned}$$

it follows at once upon substituting (10.11) and (10.12) back into (10.4) that

10.13 $$\begin{aligned} {{\mathscr {L}}}_v^{p} ({{\textsf{H}}}^{s,q}_\zeta [v]) =&~ \zeta '(t) \frac{|\nabla v|^s}{v^q} - s \zeta (t) \frac{|\nabla v|^{s-2}}{v^q} \left[ \frac{1}{2} \partial _t g + (p-1) v {\mathscr {R}ic}_f^m(g) \right] (\nabla v, \nabla v) \nonumber \\&+\zeta (t) s (p-1)\frac{|\nabla v|^{s-2}}{v^q} \left[ |\nabla v|^2 \Delta _f v - v |\nabla \nabla v|^2 - v \frac{\langle \nabla f , \nabla v \rangle ^2}{(m-n)} \right] \nonumber \\&+ \zeta (t) \frac{|\nabla v|^{s-2}}{v^{q}} \left[ s[q(p-1)+1] \langle \nabla |\nabla v|^2 , \nabla v\rangle - q[q(p-1) +p] \frac{|\nabla v|^{4}}{v}\right] \nonumber \\&- \zeta (t)\frac{s}{2v^{q-1}}(p-1) \langle \nabla |\nabla v|^{s-2} , \nabla |\nabla v|^2 \rangle + \zeta (t) s \frac{|\nabla v|^{s-2}}{v^q}\langle \nabla v, \nabla \Sigma (t,x,v) \rangle \nonumber \\&- \zeta (t)\frac{q}{v^{q+1}} |\nabla v|^s\Sigma (t,x,v)+ \Gamma '(v) [|\nabla v|^2 +\Sigma (t,x,v)] - (p-1)v\Gamma ''(v) |\nabla v|^2. \end{aligned}$$

Finally since

10.14 $$\begin{aligned} \left\langle \nabla v, \nabla \left( \frac{|\nabla v|^s}{v^q}\right) \right\rangle = \frac{s}{2} \frac{|\nabla v|^{s-2}}{v^q} \langle \nabla v, \nabla |\nabla v|^2 \rangle - q \frac{|\nabla v|^{s+2}}{v^{q+1}}, \end{aligned}$$

using (10.13) and the above it follows that for any constant $\varepsilon$ we have

10.15 $$\begin{aligned} {{\mathscr {L}}}_v^{p}({{\textsf{H}}}^{s,q}_\zeta [v])&- \varepsilon \zeta (t)\langle \nabla v, \nabla (|\nabla v|^s/v^q) \rangle \nonumber \\ =&~ \zeta '(t) \frac{|\nabla v|^s}{v^q} - s \zeta (t) \frac{|\nabla v|^{s-2}}{v^q} \left[ \frac{1}{2} \partial _t g + (p-1) v {\mathscr {R}ic}_f^m(g) \right] ( \nabla v, \nabla v) \nonumber \\&+\zeta (t) \frac{|\nabla v|^{s-2}}{v^q} \left\{ s (p-1) \left[ |\nabla v|^2 \Delta _f v - v |\nabla \nabla v|^2 - v \frac{\langle \nabla f , \nabla v \rangle ^2}{(m-n)} \right] \right. \nonumber \\&\left. + \bigg [ s[q(p-1)+1-\varepsilon /2] \langle \nabla |\nabla v|^2 , \nabla v\rangle - q[q(p-1) +p-\varepsilon ] \frac{|\nabla v|^{4}}{v}\bigg ] \right\} \nonumber \\&- \zeta (t)\frac{s}{2v^{q-1}}(p-1) \langle \nabla |\nabla v|^{s-2} , \nabla |\nabla v|^2 \rangle + \zeta (t) s \frac{|\nabla v|^{s-2}}{v^q}\langle \nabla v, \nabla \Sigma (t,x,v) \rangle \nonumber \\&- \zeta (t)\frac{q}{v^{q+1}} |\nabla v|^s\Sigma (t,x,v)+ \Gamma '(v) [|\nabla v|^2 +\Sigma (t,x,v)] - (p-1)v\Gamma ''(v) |\nabla v|^2. \end{aligned}$$

This after a rearrangement of terms gives the desired conclusion. $\square$

### Lemma 10.2

Under the assumptions of Lemma 10.1, if the metric *g* and potential *f* evolve under the flow inequality (10.1) then

10.16 $$\begin{aligned} {{\mathscr {L}}}_v^{p} ({{\textsf{H}}}^{s,q}_\zeta [v]) -&~\varepsilon \langle \nabla v, \nabla {{\textsf{H}}}^{s,q}_\zeta [v] \rangle \nonumber \\ \le&\left\{ \zeta '(t) + s \textsf{k} \zeta (t) + \zeta (t) \left[ s\Sigma _v(t,x,v) - q \frac{\Sigma (t,x,v)}{v} \right] \right\} \frac{|\nabla v|^s}{v^q} \nonumber \\&+ \frac{s\zeta (t)}{4(p-1)} \bigg [\{\varepsilon - 2[q(p-1)+1] -(p-1)\}^2 + (p-1)^2(m-1) \nonumber \\&- \frac{4(p-1)}{s} q[q(p-1) +p-\varepsilon ]\bigg ] \frac{|\nabla v|^{s+2}}{v^{q+1}} + s \zeta (t) \frac{|\nabla v|^{s-2}}{v^q}\langle \nabla v, \Sigma _x (t,x,v)\rangle \nonumber \\&- (p-1)v\Gamma ''(v) |\nabla v|^2 + \Gamma '(v) [(1-\varepsilon )|\nabla v|^2 +\Sigma (t,x,v)] . \end{aligned}$$

### Proof

We start with (10.3) in Lemma 10.1. Referring to the second and third lines on the right-hand side of the last equation (the expression with coefficient $\zeta (t) |\nabla v|^{s-2}/v^q$), suppressing $\zeta$ for now and denoting the remaining expression by $\mathbf{I}$ we have

10.17 $$\begin{aligned} \mathbf{I}=&\frac{|\nabla v|^{s-2}}{v^q} \bigg \{ s (p-1)\left[ |\nabla v|^2 \Delta _f v - v |\nabla \nabla v|^2 - v \frac{\langle \nabla f , \nabla v \rangle ^2}{(m-n)}\right] \nonumber \\&+ 2s[q(p-1)+1-\varepsilon /2] \nabla \nabla v (\nabla v, \nabla v) - q[q(p-1) +p-\varepsilon ] \frac{|\nabla v|^{4}}{v}\bigg \}\nonumber \\ =&\frac{|\nabla v|^{s-2}}{v^q} \bigg \{ \overbrace{s (p-1)\left[ |\nabla v|^2 \Delta v - v |\nabla \nabla v|^2\right] + 2s[q(p-1)+1-\varepsilon /2] \nabla \nabla v (\nabla v, \nabla v)}^{\mathbf{II} = s (p-1) [|\nabla v|^2 \textrm{tr} A - v |A|^2] + 2s [q(p-1)+1 - \varepsilon /2] |\nabla v|^2 A(e,e) } \bigg \} \nonumber \\&-s (p-1) \frac{|\nabla v|^{s-2}}{v^{q-1}} \left[ \sqrt{m-n}\frac{|\nabla v|^2}{2v} + \frac{\langle \nabla f , \nabla v \rangle }{\sqrt{m-n}} \right] ^2\nonumber \\&+ \left[ \frac{s}{4} (p-1) (m-n)- q[q(p-1) +p-\varepsilon ]\right] \frac{|\nabla v|^{s+2}}{v^{q+1}}. \end{aligned}$$

Now for the expression inside the curly brackets on the right-hand side of the second equality, say $\mathbf{II}$, setting $A=\nabla \nabla v$ with $\textrm{tr} A=\Delta v$ and $e = \nabla v/ |\nabla v|$, we can write

10.18 $$\begin{aligned} \mathbf{II}&= s (p-1) \left[ |\nabla v|^2 \Delta v - v |\nabla \nabla v|^2\right] - s\{\varepsilon -2[q(p-1)+1]\} \nabla \nabla v (\nabla v, \nabla v) \\&=-s (p-1) v |A|^2 + \left[ s (p-1)\frac{\textrm{tr} A}{|A|} - s\{\varepsilon -2[q(p-1)+1]\} \frac{A(e,e)}{|A|}\right] |\nabla v|^2 |A| \nonumber \\&=-s (p-1) v \left[ |A|^2 + \frac{|A||\nabla v|^2}{(p-1)v}\left( \{\varepsilon -2[q(p-1)+1]\} \frac{A(e,e)}{|A|}-(p-1)\frac{\textrm{tr} A}{|A|} \right) \right] , \nonumber \end{aligned}$$

or by extracting a perfect square and rearranging terms

10.19 $$\begin{aligned} \mathbf{II}=&-s (p-1) v \left[ |A| + \frac{1}{(p-1)}\left( \{\varepsilon -2[q(p-1)+1]\} \frac{A(e,e)}{|A|}-(p-1)\frac{\textrm{tr} A}{|A|}\right) \frac{|\nabla v|^2}{2v}\right] ^2\nonumber \\&+ \frac{sv}{(p-1)} \left[ \{\varepsilon -2[q(p-1)+1]\} \frac{A(e,e)}{|A|}-(p-1)\frac{\textrm{tr} A}{|A|}\right] ^2 \frac{|\nabla v|^4}{4v^2} \nonumber \\ \overbrace{\le }^{s(p-1)>0}&~\frac{s|\nabla v|^4}{4(p-1)v} \left[ \{\varepsilon -2[q(p-1)+1]\} \frac{A(e,e)}{|A|}-(p-1)\frac{\textrm{tr} A}{|A|}\right] ^2. \end{aligned}$$

Applying Lemma 7.2 to the last expression on the right with the choices of constants $a= \varepsilon -2[q(p-1)+1]$, $b= -(p-1)$ and

10.20 $$\begin{aligned} (a+b)^2 +(n-1) b^2 = \{\varepsilon - 2[q(p-1)+1] -(p-1)\}^2+ (n-1)(p-1)^2, \end{aligned}$$

then gives

10.21 $$\begin{aligned} \mathbf{II}&\le \frac{s|\nabla v|^4}{4(p-1)v} \left( \{\varepsilon -2[q(p-1)+1]\} \frac{A(e,e)}{|A|}-(p-1)\frac{\textrm{tr} A}{|A|}\right) ^2 \nonumber \\&\le \frac{s|\nabla v|^4}{4(p-1)v} \left[ \{\varepsilon - 2[q(p-1)+1] -(p-1)\}^2+ (n-1)(p-1)^2\right] . \end{aligned}$$

As a result using this inequality for $\mathbf{II}$ and substituting back into $\mathbf{I}$ in (10.17) gives

$$\begin{aligned} \mathbf{I} \le&\bigg \{ s (p-1)\left[ |\nabla v|^2 \Delta v - v |\nabla \nabla v|^2\right] + 2s[q(p-1)+1-\varepsilon /2] \nabla \nabla v (\nabla v, \nabla v) \bigg \} \frac{|\nabla v|^{s-2}}{v^q} \\&+ \left[ \frac{s}{4} (p-1) (m-n)- q[q(p-1) +p-\varepsilon ]\right] \frac{|\nabla v|^{s+2}}{v^{q+1}} \\ \le&\frac{s}{4(p-1)} \left[ \{\varepsilon - 2[q(p-1)+1] -(p-1)\}^2+ (n-1)(p-1)^2\right] \frac{|\nabla v|^{s+2}}{v^{q+1}} \\&+ \left[ \frac{s}{4} (p-1) (m-n) - q[q(p-1) +p-\varepsilon ]\right] \frac{|\nabla v|^{s+2}}{v^{q+1}} \\ \le&\bigg [ \frac{s \{\varepsilon - 2[q(p-1)+1] -(p-1)\}^2+ s(p-1)^2 (m-1)}{4(p-1)} \bigg ] \frac{|\nabla v|^{s+2}}{v^{q+1}} \\&- \bigg [ q[q(p-1) +p-\varepsilon ]\bigg ] \frac{|\nabla v|^{s+2}}{v^{q+1}}. \end{aligned}$$

Hence from (10.3) we obtain

10.22 $$\begin{aligned} {{\mathscr {L}}}_v^{p}({{\textsf{H}}}^{s,q}_\zeta [v])&- \varepsilon \zeta (t)\langle \nabla v, \nabla (|\nabla v|^s/v^q) \rangle \nonumber \\ \le&~ \zeta '(t) \frac{|\nabla v|^s}{v^q} - s \zeta (t) \frac{|\nabla v|^{s-2}}{v^q} \left[ \frac{1}{2} \partial _t g + (p-1) v {\mathscr {R}ic}_f^m(g) \right] ( \nabla v, \nabla v) \nonumber \\&+ \frac{s\zeta (t)}{4(p-1)} \bigg [\{\varepsilon - 2[q(p-1)+1] -(p-1)\}^2 + (p-1)^2(m-1) \nonumber \\&- \frac{4(p-1)}{s} q[q(p-1) +p-\varepsilon ]\bigg ] \frac{|\nabla v|^{s+2}}{v^{q+1}} - \frac{s\zeta (t)}{2v^{q-1}}(p-1) \langle \nabla |\nabla v|^{s-2} , \nabla |\nabla v|^2 \rangle \nonumber \\&+ \zeta (t) s \frac{|\nabla v|^{s-2}}{v^q}\langle \nabla v, \nabla \Sigma (t,x,v) \rangle - \zeta (t)\frac{q}{v^{q+1}} |\nabla v|^s\Sigma (t,x,v) \nonumber \\&+ \Gamma '(v) [|\nabla v|^2 +\Sigma (t,x,v)] - (p-1)v\Gamma ''(v) |\nabla v|^2. \end{aligned}$$

Finally by recalling the definition of ${{\textsf{H}}}^{s,q}_\zeta [v]$ in (10.2) we can rewrite (10.22) after a suitable rearrangement as

10.23 $$\begin{aligned} {{\mathscr {L}}}_v^{p} ({{\textsf{H}}}^{s,q}_\zeta [v])&- \varepsilon \langle \nabla v, \nabla {{\textsf{H}}}^{s,q}_\zeta [v] \rangle \nonumber \\ \le&~ \zeta '(t) \frac{|\nabla v|^s}{v^q} - s \zeta (t) \frac{|\nabla v|^{s-2}}{v^q} \left[ \frac{1}{2} \partial _t g + (p-1) v {\mathscr {R}ic}_f^m(g) \right] ( \nabla v, \nabla v) \nonumber \\&+ \frac{s\zeta (t)}{4(p-1)} \bigg [\{\varepsilon - 2[q(p-1)+1] -(p-1)\}^2 + (p-1)^2(m-1) \nonumber \\&- \frac{4(p-1)}{s} q[q(p-1) +p-\varepsilon ]\bigg ] \frac{|\nabla v|^{s+2}}{v^{q+1}} - \frac{s\zeta (t)}{2v^{q-1}}(p-1) \langle \nabla |\nabla v|^{s-2} , \nabla |\nabla v|^2 \rangle \nonumber \\&+ \zeta (t) s \frac{|\nabla v|^{s-2}}{v^q} \langle \nabla v, \nabla \Sigma (t,x,v) \rangle - \zeta (t)\frac{q}{v^{q+1}} |\nabla v|^s\Sigma (t,x,v) \nonumber \\&+ \Gamma '(v) [(1-\varepsilon ) |\nabla v|^2 +\Sigma (t,x,v)] - (p-1)v\Gamma ''(v) |\nabla v|^2. \end{aligned}$$

An application of super flow (10.1) along with $\langle \nabla v, \nabla \Sigma \rangle = \langle \nabla v, \Sigma _x \rangle + \Sigma _v |\nabla v|^2$ and the inequality $\langle \nabla |\nabla v|^{s-2}, \nabla |\nabla v|^2\rangle \ge 0$ gives the desired conclusion. Note that the last inequality follows by writing $V=|\nabla v|^2$, setting $\alpha =(s-2)/2$ (recall $s\ge 2$) and observing that $\langle \nabla V^\alpha , \nabla V \rangle = \alpha V^{\alpha -1} |\nabla V|^2 \ge 0$. $\square$

### Lemma 10.3

Under the assumptions of Lemma 10.1, if the metric *g* and potential *f* evolve under the super flow (10.1) then ${{\textsf{H}}} = \zeta (t) v^{s/[2(s-1)(p-1)]} |\nabla v|^s + \Gamma (v)$ satisfies the evolution inequality

10.24 $$\begin{aligned} {{\mathscr {L}}}_v^{p} ({{\textsf{H}}}) -&p \langle \nabla v, \nabla {{\textsf{H}}} \rangle \nonumber \\ \le&\left\{ \zeta '(t) + s \textsf{k} \zeta (t) + s \zeta (t) \left[ \Sigma _v(t,x,v)] + \frac{\Sigma (t,x,v)/v}{2(s-1)(p-1)} \right] \right\} v^\frac{s}{2(s-1)(p-1)} |\nabla v|^{s} \nonumber \\&+ s\zeta (t) \bigg [\frac{(s-1)(m-1)(p-1)^2-1}{4(s-1)(p-1)}\bigg ] v^{-1+\frac{s}{2(s-1)(p-1)}} |\nabla v|^{s+2} \nonumber \\&+ s \zeta (t) \langle \nabla v, \Sigma _x(t,x,v) \rangle v^\frac{s}{2(s-1)(p-1)} |\nabla v|^{s-2} + \Gamma '(v) \Sigma (t,x,v) \nonumber \\&- (p-1) [\Gamma '(v)+v\Gamma ''(v)] |\nabla v|^2. \end{aligned}$$

Here ${{\textsf{H}}} = {{\textsf{H}}}^{s,q}_\zeta [v]$ is as in (10.2) with $q=q^\star = - s/[2(s-1)(p-1)]$ while $s \ge 2$.

### Proof

We start with inequality (10.16) established in Lemma 10.2. Setting $\Omega (q, \varepsilon )$ to be the quadratic function

10.25 $$\begin{aligned} \Omega (q, \varepsilon ) =&~ \{\varepsilon - 2[q(p-1)+1] -(p-1)\}^2 + (p-1)^2(m-1) \nonumber \\&- \frac{4(p-1)}{s} q[q(p-1) +p-\varepsilon ], \end{aligned}$$

the latter inequality can be written as

10.26 $$\begin{aligned} {{\mathscr {L}}}_v^{p} ({{\textsf{H}}}^{s,q}_\zeta [v]) -&~\varepsilon \langle \nabla v, \nabla {{\textsf{H}}}^{s,q}_\zeta [v] \rangle \nonumber \\ \le&\left\{ \zeta '(t) + s \textsf{k} \zeta (t) + \zeta (t) \left[ s \Sigma _v(t,x,v) - q \frac{\Sigma (t,x,v)}{v} \right] \right\} \frac{|\nabla v|^s}{v^q} \nonumber \\&+ \frac{s\Omega (q,\varepsilon )\zeta (t)}{4(p-1)} \frac{|\nabla v|^{s+2}}{v^{q+1}} + s \zeta (t) \frac{|\nabla v|^{s-2}}{v^q}\langle \nabla v, \Sigma _x (t,x,v)\rangle \nonumber \\&- (p-1)v\Gamma ''(v) |\nabla v|^2 + \Gamma '(v) [(1-\varepsilon )|\nabla v|^2 +\Sigma (t,x,v)]. \end{aligned}$$

A straightforward optimisation of $\Omega$ leads to the optimiser $(q^\star , \varepsilon ^\star )$ with

10.27 $$\begin{aligned} q^\star = - \frac{s}{2(s-1)(p-1)} , \qquad \text {and} \qquad \varepsilon ^\star = p, \end{aligned}$$

and subsequently the optimal value

10.28 $$\begin{aligned} \Omega (q^\star , \varepsilon ^\star ) =-\frac{1}{s-1} +(p-1)^2(m-1). \end{aligned}$$

Substituting the values (10.27) and (10.28) back into (10.26) gives the desired inequality $\square$

### Remark 10.4

The value $\Omega (q^\star , \varepsilon ^\star )$ is non-positive *iff* $1<p<1+1/\sqrt{(s-1)(m-1)}$ as can be easily seen from (10.28). The importance of this non-positive sign will become apparent in the next lemmas where we apply maximum principle.

### Corollary 10.5

Let $(\mathscr M,g,e^{-f} dv_g)$ be a smooth metric measure space with $\mathscr M$ closed. Let *u* be a positive smooth solution to (1.1) where $1<p \le 1+1/\sqrt{(s-1)(m-1)}$ with $s \ge 2$ and set $v =pu^{p-1}/(p-1)$. Assume the metric $g$ and potential $f$ satisfy the super flow inequality (10.1). Moreover suppose the following conditions hold for some *a*:

- $$\Gamma '(v) \Sigma (t,x,v) \le 0$$,
- $$\Gamma '(v) + v \Gamma ''(v) \ge 0$$,
- $$\langle \nabla v, \Sigma _x(t,x,v) \rangle \le 0$$,
- $$\Sigma _v(t,x,v) + \Sigma (t,x,v)/[2(s-1)(p-1)v] \le a$$.

Then for all $x \in \mathscr M$ and $0<t \le T$ we have

10.29 $$\begin{aligned} {[}v^\frac{s}{2(s-1)(p-1)} |\nabla v|^s] (x,t) \le e^{s(\textsf{k}+a)t} \left\{ \max _{\mathscr M} \left[ v^\frac{s}{2(s-1)(p-1)} |\nabla v|^s + \Gamma (v) \right] _{t=0} - \Gamma (v(x,t)) \right\} . \end{aligned}$$

### Proof

Using (10.24) and the assumptions on $\Gamma$ and $\Sigma$ in the statement of the corollary, we can write for ${{\textsf{H}}} = \zeta (t) v^{s/[2(s-1)(p-1)]} |\nabla v|^s + \Gamma (v)$

10.30 $$\begin{aligned} {{\mathscr {L}}}_v^{p}&({{\textsf{H}}}) - p \langle \nabla v, \nabla {{\textsf{H}}} \rangle \nonumber \\ \le&\left\{ \zeta '(t) + s\textsf{k} \zeta (t) + s \zeta (t) \left[ \Sigma _v(t,x,v)] + \frac{\Sigma (t,x,v)/v}{2(s-1)(p-1)}\right] \right\} v^\frac{s}{2(s-1)(p-1)} |\nabla v|^{s} \nonumber \\&+ s\zeta (t) \bigg [\frac{(s-1)(m-1)(p-1)^2-1}{4(s-1)(p-1)}\bigg ] v^{-1+ \frac{s}{2(s-1)(p-1)}} |\nabla v|^{s+2} \nonumber \\&+ s \zeta (t) \langle \nabla v, \Sigma _x(t,x,v) \rangle v^\frac{s}{2(s-1)(p-1)} |\nabla v|^{s-2} + \Gamma '(v)\Sigma (t,x,v) \nonumber \\&-(p-1)[\Gamma '(v)+v\Gamma ''(v)] |\nabla v|^2 \nonumber \\ \le&~ \left[ \zeta '(t) + s({{\textsf{k}}}+a) \zeta (t)\right] v^\frac{s}{2(s-1)(p-1)}|\nabla v|^{s} \nonumber \\&+ s\zeta (t) \bigg [\frac{(s-1)(m-1)(p-1)^2-1}{4(s-1)(p-1)}\bigg ] v^{-1+ \frac{s}{2(s-1)(p-1)}} |\nabla v|^{s+2}. \end{aligned}$$

The function $\zeta (t) = e^{-s(\textsf{k}+a)t}$ is non-negative, smooth and satisfies $\zeta '+s(\textsf{k}+a)\zeta =0$. Thus substituting in (10.30) and noting $(s-1)(m-1)(p-1)^2 \le 1$ due to the range of *p* we have

10.31 $$\begin{aligned} {{\mathscr {L}}}_v^{p} ({{\textsf{H}}}) - p \langle \nabla v, \nabla {{\textsf{H}}} \rangle \le 0. \end{aligned}$$

The assertion is now a consequence of the weak maximum principle giving

10.32 $$\begin{aligned} {{\textsf{H}}}(x,t)&= e^{-s(\textsf{k}+a)t} [v^\frac{s}{2(s-1)(p-1)} |\nabla v|^s](x,t) + \Gamma (v(x,t)) \nonumber \\&\le \max _{\mathscr M} [v^\frac{s}{2(s-1)(p-1)} |\nabla v|^s + \Gamma (v)]_{t=0} = \max _{\mathscr M} {{\textsf{H}}}(x,0). \end{aligned}$$

The proof is thus complete. $\square$

### Corollary 10.6

Let $(\mathscr M,g,e^{-f} dv_g)$ be a smooth metric measure space with $\mathscr M$ closed. Let *u* be a positive smooth solution to (1.1) where $1<p \le 1+1/\sqrt{m-1}$ and set $v =pu^{p-1}/(p-1)$. Assume the metric $g$ and potential $f$ satisfy the super flow inequality (10.1) with $\textsf{k} \ge 0$. Moreover suppose the following conditions hold:

- $$\Sigma (t,x,v) \le 0$$,
- $$\langle \nabla v, \Sigma _x(t,x,v) \rangle \le 0$$,
- $$\Sigma _v(t,x,v) + \Sigma (t,x,v)/[2(p-1)v] \le 0$$.

Then for $x \in \mathscr M$ and $0<t \le T$ we have

10.33 $$\begin{aligned} \frac{p^2t}{1+2{{\textsf{k}}}t} [v^\frac{1}{p-1} |\nabla v|^2](x,t) \le (p-1) \left[ \max _{\mathscr M} v^\frac{p}{p-1}(x,0) - v^\frac{p}{p-1}(x,t) \right] . \end{aligned}$$

### Proof

For ${{\textsf{H}}}[v] = \zeta (t) v^{1/(p-1)} |\nabla v|^2 + (p-1)v^{p/(p-1)}/p^2$ where $\Gamma (x,v)=(p-1)v^{p/(p-1)}/p^2$, $s=2$ and $q^\star =-1/(p-1)$, we have from Lemma 10.3,

10.34 $$\begin{aligned} {{\mathscr {L}}}_v^{p} ({{\textsf{H}}}) -&~p \langle \nabla v, \nabla {{\textsf{H}}} \rangle \nonumber \\ \le&\left\{ \zeta '(t) + 2\textsf{k} \zeta (t) + 2 \zeta (t) \left[ \Sigma _v(t,x,v)] + \frac{\Sigma (t,x,v)}{2(p-1)v} \right] \right\} v^\frac{1}{p-1} |\nabla v|^2 \nonumber \\&+ \frac{(m-1)(p-1)^2-1}{2(p-1)} \zeta (t) v^\frac{2-p}{p-1} |\nabla v|^4 + 2 \zeta (t) \langle \nabla v, \Sigma _x(t,x,v) \rangle v^\frac{1}{p-1} \nonumber \\&+ \Gamma '(v) \Sigma (t,x,v) -(p-1) [\Gamma '(v)+v\Gamma ''(v)] |\nabla v|^2 \nonumber \\ \le&\left\{ \zeta '(t) + 2\textsf{k} \zeta (t) + 2 \zeta (t) \left[ \Sigma _v(t,x,v)] + \frac{\Sigma (t,x,v)}{2(p-1)v} \right] \right\} v^\frac{1}{p-1} |\nabla v|^2 \nonumber \\&+ \frac{(m-1)(p-1)^2-1}{2(p-1)} \zeta (t) v^\frac{2-p}{p-1} |\nabla v|^4 + 2 \zeta (t) \langle \nabla v, \Sigma _x(t,x,v) \rangle v^\frac{1}{p-1} \nonumber \\&+ \frac{1}{p} v^\frac{1}{p-1} \Sigma (t,x,v) - v^\frac{1}{p-1} |\nabla v|^2. \end{aligned}$$

In particular, with the assumptions in the statement of the corollary this gives

10.35 $$\begin{aligned} {{\mathscr {L}}}_v^{p} ({{\textsf{H}}}) - p \langle \nabla v, \nabla {{\textsf{H}}} \rangle \le&~[\zeta '(t) + 2\textsf{k} \zeta (t) -1] v^\frac{1}{p-1} |\nabla v|^2 \nonumber \\&~+ \frac{(m-1)(p-1)^2-1}{2(p-1)} \zeta (t) v^\frac{2-p}{p-1} |\nabla v|^4. \end{aligned}$$

Now when $\textsf{k} \ge 0$ by taking $\zeta (t) = t/(1+2\textsf{k} t)$ we have $(\zeta ' + 2 \textsf{k} \zeta -1) \le 0$. Therefore subject to $1<p \le 1+1/\sqrt{m-1}$ we have ${{\mathscr {L}}}_v^{p}({{\textsf{H}}}) - p \langle \nabla v, \nabla {{\textsf{H}}} \rangle \le 0$. The conclusion now follows by an application of the weak maximum principle. $\square$
